# Multi-Gene Expression Predictors of Single Drug Responses to Adjuvant Chemotherapy in Ovarian Carcinoma: Predicting Platinum Resistance

**DOI:** 10.1371/journal.pone.0030550

**Published:** 2012-02-10

**Authors:** J. Stuart Ferriss, Youngchul Kim, Linda Duska, Michael Birrer, Douglas A. Levine, Christopher Moskaluk, Dan Theodorescu, Jae K. Lee

**Affiliations:** 1 Department of Obstetrics, Gynecology and Reproductive Sciences, Temple University, Philadelphia, Pennsylvania, United States of America; 2 Division of Biostatistics and Epidemiology, Department of Public Health Sciences, University of Virginia, Charlottesville, Virginia, United States of America; 3 Thornton Gynecologic Oncology Division, Department of Obstetrics and Gynecology, University of Virginia, Charlottesville, Virginia, United States of America; 4 Department of Medicine, Harvard Medical School, Boston, Massachusetts, United States of America; 5 Gynecology Service and Department of Surgery, Memorial Sloan-Kettering Cancer Center, New York, New York, United States of America; 6 Department of Pathology, University of Virginia, Charlottesville, Virginia, United States of America; 7 Department of Surgery and Pharmacology, University of Colorado at Denver, Aurora, Colorado, United States of America; Vanderbilt University Medical Center, United States of America

## Abstract

Despite advances in radical surgery and chemotherapy delivery, ovarian cancer is the most lethal gynecologic malignancy. Standard therapy includes treatment with platinum-based combination chemotherapies yet there is no biomarker model to predict their responses to these agents. We here have developed and independently tested our multi-gene molecular predictors for forecasting patients' responses to individual drugs on a cohort of 55 ovarian cancer patients. To independently validate these molecular predictors, we performed microarray profiling on FFPE tumor samples of 55 ovarian cancer patients (UVA-55) treated with platinum-based adjuvant chemotherapy. Genome-wide chemosensitivity biomarkers were initially discovered from the *in vitro* drug activities and genomic expression data for carboplatin and paclitaxel, respectively. Multivariate predictors were trained with the cell line data and then evaluated with a historical patient cohort. For the UVA-55 cohort, the carboplatin, taxol, and combination predictors significantly stratified responder patients and non-responder patients (p = 0.019, 0.04, 0.014) with sensitivity = 91%, 96%, 93 and NPV = 57%, 67%, 67% in pathologic clinical response. The combination predictor also demonstrated a significant survival difference between predicted responders and non-responders with a median survival of 55.4 months vs. 32.1 months. Thus, COXEN single- and combination-drug predictors successfully stratified platinum resistance and taxane response in an independent cohort of ovarian cancer patients based on their FFPE tumor samples.

## Introduction

Epithelial ovarian cancer (EOC) is responsible for more deaths of women in the United States than any other gynecologic malignancy [1]. Despite attempts to implement effective early detection, the majority of women continue to present with advanced stage disease. While over 70% of patients will achieve a complete response with primary cytoreductive surgery and platinum-based chemotherapy, nearly 75% will recur in an average of 21 months [2]. In the recurrent setting, ovarian cancer is rarely cured, in large part due to progressive chemoresistance. For this reason, the 5-year overall survival for EOC remains around 20% [3].

Primary surgery with maximal cytoreduction followed by adjuvant chemotherapy with a platinum-taxane doublet is the initial treatment of choice in advanced ovarian cancer. Despite prospective data demonstrating a more favorable outcome for patients who had complete surgical removal of metastatic disease, the majority of patients continue to experience recurrence, especially when a patient's tumor is platinum-resistant [2]. Therefore, it is well recognized that surgery alone cannot overcome disease progression in ovarian cancer, and the phenotype of “platinum resistance” is thus unarguably one of the most important clinical determinates [4]. About 30% of patients whose tumors are platinum-resistant will generally either progress during primary therapy or shortly thereafter—a grim reminder of the limits of current ovarian cancer care and the need for improved understanding of tumor biology and therapeutics over surgical success. Moreover, the setting of platinum resistance is a clinical conundrum: even though multiple FDA-approved chemotherapy agents are available for treatment of recurrent ovarian cancer, all have similar clinical response rates and thus there is no preferred standard second-line chemotherapy to offer these patients. Furthermore, no diagnostic tool or guidance is available to provide individualized care for the heterogeneous group of patients who comprise current clinical practice.

Recent efforts to improve survival after primary therapy have focused on novel combinations of standard chemotherapies and the use of targeted agents as seen in two recent Gynecologic Oncology Group trials: GOG182 and GOG218 [2], [5]. An alternative approach has been to focus on determining the sensitivity of an individual patient's tumor to standard medicines with a variety of drug sensitivity and resistance tests [6], [7]. The underlying premise of this approach is that by matching a given chemotherapy to an individual tumor with demonstrated sensitivity, physicians hope to achieve higher response rates, more durable tumor-free intervals, and fewer side effects compared to the standard of care (e.g. empiric choice of agents for individual patients). Unfortunately, when examined in a randomized trial, these chemotherapy sensitivity and resistance tests did not improve progression free or overall survival compared to the standard of care [8].

Molecular prediction signatures have also been developed using retrospective data sets of ovarian cancer patients treated with platinum-based chemotherapy [9]. However, these current methods for assessing chemosensitivity have been of limited use due to several shortcomings. First, these tests and signatures have been developed only based on patient data restricted to current drug combinations. Consequently, these patient-based predictors were unable to differentiate tumors with heterogeneous responses to various single agents. Also, previous molecular prediction techniques were validated based on highly-controlled patient cohorts in clinical trials that collected fresh frozen tissues. These techniques often fail to consistently perform well with lower-quality patient samples such as FFPE tissues that are customarily collected in clinical practice. Because of these limitations, molecular prediction signatures have been neither validated against a vast amount of archived FFPE patient samples, nor readily applied in diverse clinical settings.

The co-expression extrapolation (COXEN) method, an *in vitro* cell-line-based multi-gene prediction technique, has been demonstrated previously with its high potential to forecast chemotherapeutic outcomes of cancer patients [10]. Several subsequent studies have provided promising results in different cancer sites including breast, ovarian, and bladder cancer [11], [12], [13]. COXEN predictors can x initially be developed independently from patient tumors that are often treated with various drug combinations, by using a single chemotherapeutic agent's *in vitro* cancer cell-line activities associated with genome-wide expression data. The so-called COXEN biomarkers that are concordantly regulated between the cell lines and *in vivo* patient tumors are then further identified from these initial biomarkers to link *in vitro* cell line chemosensitivity to a patient's chemotherapeutic response, overcoming the differences of the tumor microenvironment and drug metabolism [13].

Since nearly all EOC tumors (>70%) will be platinum-sensitive during primary therapy, only the identification of patients sensitive to platinum agents may not provide high clinical utility. A more clinically useful scenario would be the reliable identification of patients for whom standard therapies will fail (i.e. the small proportion (less than 30%) of platinum-resistant patients). These patients could then be guided to alternative chemotherapy agents and treatment options, potentially avoiding unnecessary toxicity. We undertook this study in an effort to validate the COXEN prediction assays for their clinical utility, using an independent ovarian cancer patient cohort with archived FFPE tumor samples. Our hypothesis was that COXEN could reliably predict platinum resistance in a series of advanced epithelial ovarian cancer patients.

## Materials and Methods

### Cell line drug activity and microarray data

*In vitro* drug activity and microarray data of the NCI-60 cancer cell panel were previously described elsewhere [10]. In brief, publicly-available drug sensitivity data, expressed in terms of 50% growth inhibition (GI50) for the NCI-60 were obtained from the NCI DTP web site (http://dtp.nci.nih.gov). NCI-60 expression profiling data on HG-U133A GeneChip® arrays (Affymetrix, Santa Clara, CA) were from a public domain at the National Cancer Institute (http://discover.nci.nih.gov). The second cell line set, Peter-18, was based on six ovarian cancer cell lines with *in vitro* drug activity for carboplatin [14]. These cell lines were originally derived from six ovarian carcinoma (papillary serous adenocarcinoma) patients. These cell lines were then characterized as either carboplatin sensitive (n = 3) or resistant (n = 3) based on their *in vitro* cell-line drug sensitivity after treatment. Specifically, these cell lines were experimented to evaluate the cytotoxic index (% kill) of ∼350 cells seeded into 60-well microtiter plates, grown for 24 hrs and treated with different concentrations of carboplatin, compared to untreated controls at 48 hrs after treatment [14]. Three replicated cultures of each cell line were then subject to genome-wide expression profiling using Affymetrix HG-U95A GeneChip® arrays. These sets are summarized in **Table 1**.

**Table 1:** Cell and patient data sets used for COXEN Predictor Training and Testing.

*Name*	*Sample type*	*Array Platform* *(# of probes)*	*Responder* *(sensitive)*	*Non-responder* *(resistant)*	*Drugs*
**(Training)**					
**NCI-60**	Cell lines	HG-U133A(22,215)	10	22	Taxol
**Peter-18** **(GSE1926)**	Cell lines	HG-U95(9,530)	9	9	Carboplatin
**Bonome-185**	Humanpatients	HG-U133A(22,283)	112	55	Carboplatin, Taxol, Cisplatin, Cytoxan
**(Testing)**					
**Dressman-119**	Humanpatients	HG-U133A(22,215)	85	34	Platinum-based chemotherapy
**UVA-55**	Humanpatients	HG-U133+2(54,675)	‵32	23	Carboplatin Taxol

NCI-60 and Peter-18 cell-line data sets were used to discover chemosensitivity biomarkers and to train multivariate statistical prediction models for paclitaxel and carboplatin, respectively. Bonome-185 set was used to select the biomarkers with the consistent directions of differential expression. Dressman-119 set was used to independently evaluate the trained predictors and to derive the optimal cutoff value of each predictor. UVA-55 set was purely used to test the predictability of the COXEN predictors in a prospective manner.

### Historical patient sets for predictor development and evaluation

Microarray gene expression data from frozen tissue samples obtained at the time of primary cytoreductive surgery from two previously-published human ovarian cancer cohorts were also used for the development and independent evaluation of our molecular predictors. The first cohort of 185 primary ovarian tumors treated with adjuvant chemotherapy was originally obtained for identifying prognostic molecular signatures of survival [15]. We used the subset of 167 patients with platinum-based chemotherapeutic response information for our predictor development. These patients comprised 112 (67%) complete response (CR), 41 (25%) partial response (PR), and 14 (8%) progress of disease (PD). The second set (Dressman-119) of 119 ovarian cancer patients from the Duke University and H. Lee Moffitt Cancer Center also received platinum-based adjuvant chemotherapy [9]. Of 119, 85 (71%) patients had a complete response whereas 34 (29%) patients showed an incomplete response (IR) to the chemotherapy. Expression profiling data of the frozen-tissue tumor samples from both sets were available with Affymetrix HG-U133A GeneChip® arrays.

### Independent cohort of patients and FFPE tumor samples

After an Institutional Review Board approval, the UVA Cancer Registry was queried to identify stage III–IV epithelial ovarian cancer patients treated between 1995–2004 whose follow-up information was available for at least five years or were deceased. For inclusion in the validation cohort, patients must have had a primary surgery followed by platinum-based chemotherapy. Sixty-five patients were identified both from the UVA Cancer Registry and Biorepository Tissue Research Facility (BTRF). Patients were excluded if they lacked adequate follow-up clinical data for review. These cases were also examined whether they had adequate archived pathologic material for molecular analysis. Ten patients were excluded from these, leaving 55 patients available for the validation of COXEN predictors (UVA-55). Formalin-fixed paraffin embedded (FFPE) tissue blocks were obtained for each patient in the validation cohort. These blocks were reviewed by a pathologist to ensure an adequate tumor was present for analysis. The corresponding histologic sections were examined by a pathologist and areas of tissue with tumor cell percentages >70% were selected. Only blocks that contained >2 mm of tissue thickness were used to obtain tumor tissue of the block with 3 mm biopsy punches for subsequent microarray analysis.

The detailed clinical characteristics of the cohort are summarized in **Table 2**. All patients received a primary surgical effort by a board-certified gynecologic oncologist and a gynecologic pathologist reviewed all pathology. Adjuvant chemotherapy consisted of platinum (carboplatin or cisplatin) either alone (n = 2) or in combination with a taxane (n = 51, paclitaxel or docetaxel) or cyclophosphamide (n = 2). Response assessment and surveillance schedules followed accepted clinical practice. The median age in this cohort was 62 (range 38–65) and the majority were stage III cancers (91%) of serous histology (85%). The median progression-free survival was 13 months (95% CI 10–16) and the median overall survival for the cohort was 50 months (95% CI 32–68). All research involving human participants have been approved by the institutional review board at the University of Virginia. Witten informed consents were obtained from all participants involved in the study.

**Table 2:** Clinical Characteristics of the UVA-55 Cohort.

*Characteristic*	*N (%)*
Patients	55
Median Age (range)	62 (38–65)
Ethnicity	
White	51 (93%)
Black	4 (7%)
Stage	
III	50 (91%)
IV	5 (9%)
Histology	
Serous	47 (85%)
Clear Cell	5 (9%)
Other	3 (6%)
Surgical Outcome	
Optimal (<1 cm)	30 (55%)
Sub-optimal (≥1 cm)	25 (45%)
Response to Initial Therapy	
CR	32 (58%)
PR, PD	23 (42%)
Recurrences	48 (87%)
Deaths	36 (65%)
Survival (months)	
Median PFS	13 (95% CI, 10–16)
Median OS	50 (95% CI, 32–68)

CR = Complete Response, PR = Partial Response, PD = Progressive Disease, PFS = Progression Free Survival, OS = Overall Survival, CI = Confidence Interval.

### Microarray Profiling on FFPE Tumor Samples

Gene expression profiling from the two 3 mm core punches of FFPE tumor tissue blocks containing >70% tumor cells were performed by Almac Diagnostics, Inc. (Durham, NC) based on its standard protocol. In particular, RNA extraction and amplification were performed with the NuGen WT-Ovation™ FFPE RNA Amplification kit which is specialized to overcome the cross-linked and fragmented RNAs in FFPE samples (NuGen, Inc., San Carlos, CA). Data normalization and quality control assessment of the hybridization results were performed with the RMA Bioconductor package (http://www.bioconductor.org).

### Statistical Methods

**Figure 1:**
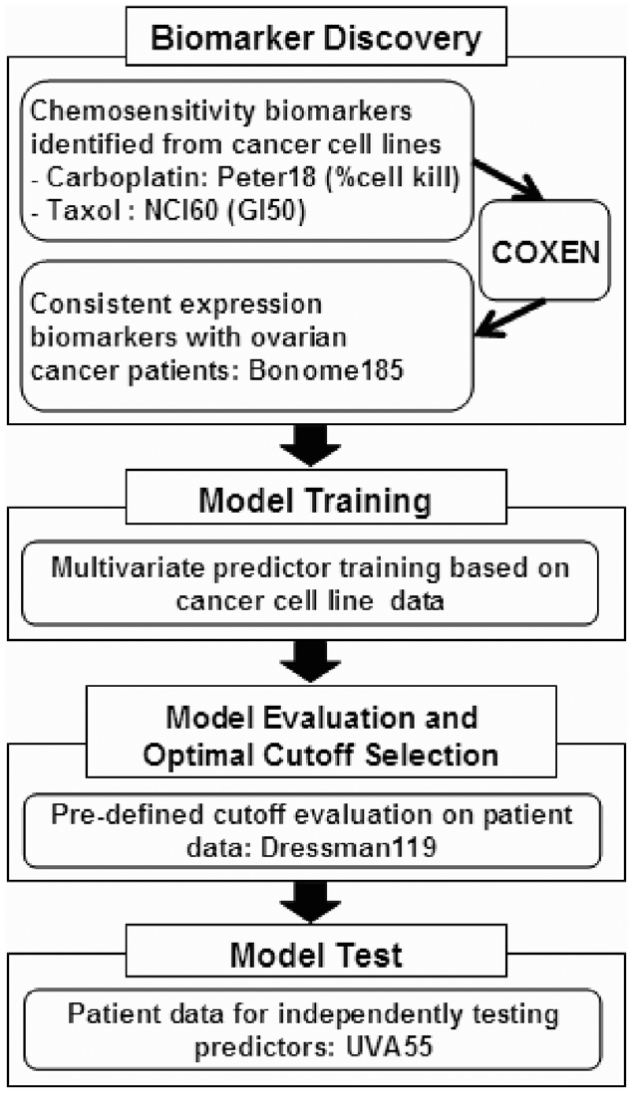
Schematic Summary of COXEN Predictor Development and Test.

The procedures for our predictor training and test are summarized in **Figure 1**. In brief, COXEN predictors for paclitaxel and carboplatin were first derived from *in vitro* drug sensitivity and microarray data [10]. Candidate biomarkers that were highly associated with carboplatin and paclitaxel sensitivity were identified from the Peter-18 and NCI-60 microarray data, respectively. That is, the 10–35% most and least sensitive cell lines based on GI50 (growth-inhibition 50%) or cell kill percent values were correlated with genome-wide expression data to identify initial chemosensitivity biomarkers for each drug. These initial biomarkers were filtered with FFPE-robust probe sets (about 55% of all probe sets) which were previously derived from our in-house dataset comprising >27 paired frozen and FFPE tumor samples (data not shown). These FFPE-robust chemosensitivity biomarkers were then triaged based on the COXEN coefficient which represents the degree of concordance of expression regulation between the NCI-60 cell lines and human ovarian cancer patients. In brief, the mathematical derivation of COXEN coefficient is based on the so-called “correlation of correlations,” which first calculates the expression correlations within each set on the identical set of genes of interest for both sets and then evaluates gene-by-gene correlation between the two correlation matrices of the two sets. This kind of 2nd-order correlation has proven useful to investigate various gene networks to identify concordant ones across different data sets by us and others [16], [17], [18]. More detailed description of the COXEN algorithm has been desctibed elsewhere [10], [11]. Expression patterns of these NCI-60-based chemosensitivity biomarkers were further compared to those in a cohort of 185 human ovarian cancer patients, and were excluded if their expression changes were inconsistent between the two sets. Some relevant issues on these filtering steps are discussed later.

Genes with significant COXEN coefficients were then used for our drug-specific prediction modeling using a double cross-validated linear discriminant analysis (LDA) on the Peter-18 cell lines for carboplatin and the NCI-60 cell lines for paclitaxel, respectively as described elsewhere [19]. The resulting COXEN predictors were simultaneously applied to the two independent patient cohorts of Dressman-119 frozen and UVA-55 FFPE tumor samples. Assuming their independence, combined prediction scores from the two individual drug predictors were simply calculated to generate a combination chemotherapy (CT) predictor.

Performance of these predictors was first evaluated by testing a significant difference in the COXEN scores between the CR and PR patient groups using a non-parametric Wilcoxon rank-sum test. We also performed an ROC (receiver operator characteristics) analysis both to evaluate their overall predictability by the area under the curve (AUC) and to define optimal cutoff values for high clinical utility. The optimal cutoff values for the COXEN predictors were first determined by maximizing the Youden index ( = sensitivity+specificity-1) on the ROC curves. At this Youden cutoff value, the sensitivity, specificity, positive predictive value (PPV), and negative predictive value (NPV) for stratifying clinical responders (pCRs) from non-responders were then derived independently on our validation patient sets [20]. However, if these mathematically-derived cutoff values could not provide a high clinical utility, i.e., a low NPV, we then found alternative cutoff values by maximizing NPV (see Supplementary Methods in Text S1 for more details).

## Results

### COXEN Predictors of Carboplatin and Paclitaxel

**Figure 2:**
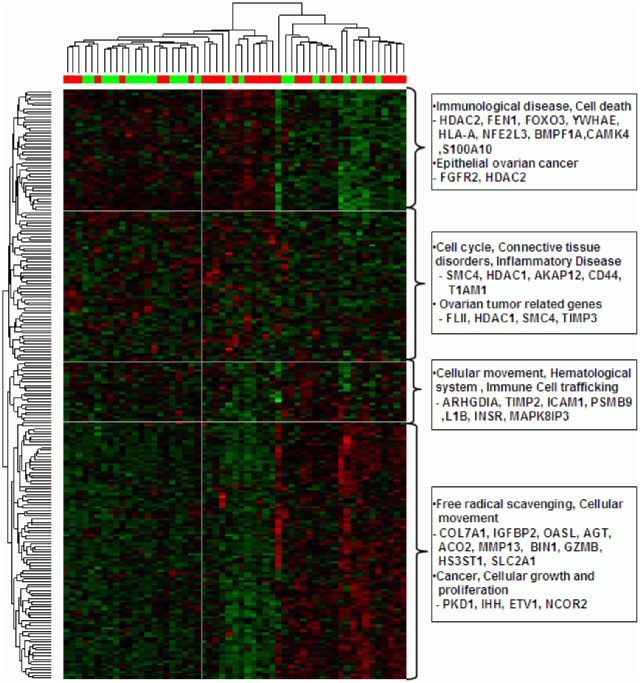
COXEN Biomarkers and Gene Networks for Carboplatin. Clustering heatmap analysis with major gene networks with x-axis responder (red) and non-responder (green) patients and y-axis Immunological disease/cell death entwork (red), Cell cycle/Connective tissue disorders/Inflammator disease network (green), Cellular movement/Hematological system/Immune cell trafficking network (yellow), and Free radical scavenging/cellular movement/cancer/cellular growth and proliferation network (blue).

We identified the final FFPE-robust 251 and 125 biomarkers for the training of carboplatin and paclitaxel predictors, respectively, on the NCI-60 and Peter-18 cell line panels. For each set of biomarkers, hierarchical clustering and biological pathway analyses were performed, the latter by Ingenuity Pathway Analysis (IPA, Ingenuity, Inc., Redwood City, CA; **Supplementary Figure S1**). Distinctly, carboplatin biomarkers were from cell cycle/tissue disorder, hematological system development, organismal functions, and cellular growth/proliferation associated network functions. Biomarkers from these networks were, in fact, found to be closely clustered in the clustering heatmap analysis (**Figure 2**; Supplementary **Table S1**). In particular, the responders (red) and nonresponders (green) were found to be generally clustered together even in this unsupervised clustering analysis. Paclitaxel biomarkers were also found to be from cell death, DNA replication, recombination, and repair networks (Supplementary **Table S1; Supplementary Figure S1B, C**). These biomarkers of each drug were used to develop COXEN multivariate prediction models.

### Validation on independent patient cohorts

**Figure 3:**
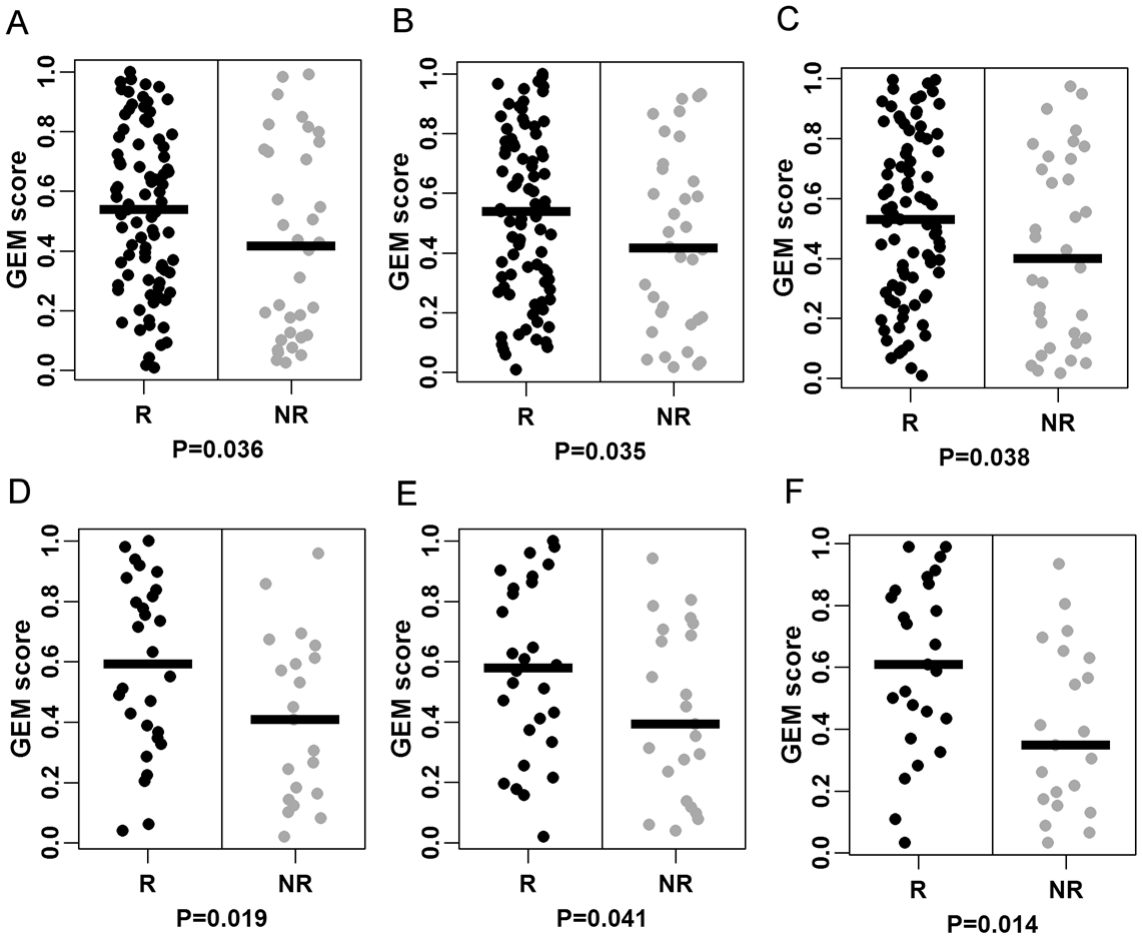
Evaluation and Validation result on ovarian patients. **Figures 3**A, 3B, and 3C are evaluation resulton the Dressman-119 cohort; (A) the distribution of COXEN scores for Carboplatin; (B) COXEN scores for paclitaxel; (C) COXEN scores for the drug combination of Carboplatin and Paclitaxel. Figures 3D, 3E, and 3F are validation result on the UVA-55 cohort for Carboplatin, Paclitaxel, and for the drug combination of Carboplatin and Paclitaxel, respectively. Coxen scores of responder (black) and non-responder(gray). P-values calculated by Wilcoxon rank sum test.

The performance of these multi-gene predictors was first independently examined on the frozen-tissue-based Dressman-119 cohort. We found the COXEN scores of both carboplatin and paclitaxel predictors for responder patients were significantly higher than those of non-responder patients in this set (Wilcoxon rank-sum test P = 0.036 for carboplatin and P = 0.035 for paclitaxel). The combined prediction scores for the two drugs also significantly stratified responder patients from the non-responders in this cohort (Wilcoxon rank-sum test P = 0.038) (**Figure 3A, B, and C**).

The unaltered COXEN predictors were then used for predicting chemotherapeutic responses of the FFPE tissue-based UVA-55 cohort for both single and combination agents. We again found the predicted sensitivity scores of each of carboplatin and paclitaxel of the responders were significantly higher than those of non-responders in the UVA adjuvant patients (Wilcoxon rank-sum test P = 0.019 for carboplatin and P = 0.041 for paclitaxel). The combination-drug predictor also provided a significant difference between responders and non-responders (P = 0.014) in the UVA-55 cohort (**Figure 3D, E, and F**). These predictors provided high stratification capability between responders and non-responders in the ROC analysis: combination predictor AUC 0.604 [95% CI: 0.483–0.723] and 0.703 [0.549–0.856] with Wilcoxon P = 0.038 and 0.009 on the two sets, respectively, which well demonstrated the significance of their overall predictability (**Table 3**).

**Table 3:** Prediction performance of COXEN predictors.

Compound	Data(Res, Nonres)	*AUC* *[95% CI]* *(P-value)*	Sensitivity	(Specificity)	PPV	NPV
**Carboplatin**	Dressman(85, 34)	0.606[0.483–0.730](p = 0.036)	0.941(80/85)(0.891–0.991)	0.294 (10/34)(0.141–0.447)	0.769(80/104)(0.688–0.850)	0.667 (10/15)(0.428–0.905)
	UVA-55(32, 23)	0.617[0.464–0.769](p = 0.072)	0.906 (29/32)(0.805–1)	0.174 (4/23)(0.019–0.328)	0.604 (29/48)(0.465–0.743)	0.571 (4/7)(0.205–0.938)
**Taxol**	Dressman(85, 34)	0.595[0.478–0.712](p = 0.053)	0.976 (83/85)(0.944–1)	0.176 (6/34)(0.048–0.304)	0.747 (83/111)(0.667–0.828)	0.75 (6/8)(0.450–1)
	UVA-55(28, 23)	0.642[0.488–0.797](p = 0.041)	0.964 (27/28)(0.900–1)	0.087 (3/23)(0.422–0.702)	0.562 (27/48)(0.422–0.703)	0.667 (2/3)(0.133–1)
**Carbo/Tax**	Dressman(85, 34)	0.604[0.483–0.723](p = 0.038)	0.976 (83/85)(0.944–1)	0.147 (5/34)(0.028–0.266)	0.741 (83/112)(0.660–0.822)	0.714 (5/7)(0.380–1)
	UVA-55(28, 23)	0.703[0.549–0.856](p = 0.009)	0.928 (26/28)(0.833–1)	0.174 (4/23)(0.019–0.329)	0.577 (26/45)(0.433–0.722)	0.667 (4/6)(0.289–1)

The overall predictability (AUC) of identical COXEN predictors are summarized on the Dressman-119 and UVA-55 cohorts by AUC values with their 95% CIs and p-values. Cutoff values of COXEN predictors were derived by maximizing NPVs on the Dressman-119 cohort. Sensitivity, specificity, PPV, and NPV values were evaluated on both Dresseman-119 and independent UVA-55 cohort.

### Survival benefit of COXEN predictors

**Figure 4:**
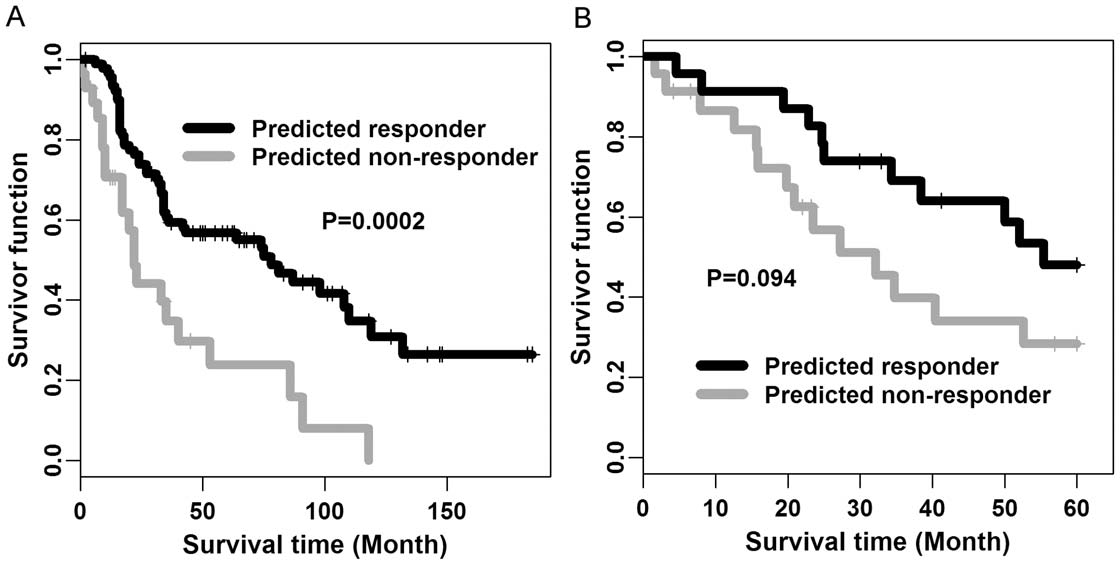
Overall Survival Difference between COXEN Predicted Responders vs. Non-Responders. (A) Kaplan Meier survival plot of Dressman-119 cohort. (B) Kaplan Meier survival plot of UVA-55 cohort. The survival curves of patients predicted to be responders (Red) and non-responders (Green) showed significant differences between COXEN predicted responders and non-responders with median survival times 77.8 and 22.3 months for the Dressman-119 cohort and 55.4 and 32.2 months for the UVA-55 cohort between the two groups. P-values were calculated by Log-rank test.

As described above, these predictors showed statistical significance in their overall predictability. However, one still needs to define a fixed cutoff value for each predictor in order to evaluate its clinical benefit *a priori*, thus defining responder vs. nonresponder in a future patient set at this cutoff. We first chose the mathematical cutoff points of these predictors by maximizing the Youden index ( = specificity+sensitivity-1) on the Dressman-119 cohort cohort (**Supplementary Table S2**). These predictors with the Youden cutoff values (for stratifying pathologic clinical response to chemotherapy) provided a significant survival difference between predicted responder and non-responder groups. Only overall survival time was available both for Dressman-119 and UVA-55 cohorts so we used this survival outcome endpoint to directly compare predicted survival benefit on the two cohorts. Overall survival time longer than 5 years was censored to avoid mathematical artifacts from a few patients' outlying survival times (e.g. >10 years) in our survival analysis. Death beyond this time period (after chemotherapy) may not be directly relevant to the chemotherapeutic response. The clinical characteristics (e.g. surgical outcome, chemotherapy agents) of patients with >5 years of survival were not significantly different from the remaining cohort (data not shown). For the Dressman-119 cohort, we found the combination-drug COXEN predictor provided a highly significant survival difference by a Kaplan-Meier survival analysis (log-rank test P = 0.0002; **Figure 4A**). The median survival times were 77.8 and 22.3 months between predicted responder and non-responder groups in the cohort. For the UVA-55 cohort, the identical combination-drug predictor also similarly provided a survival difference between the two groups (log-rank test P = 0.094; **Figure 4B**); statistical significance was only marginally significant, likely due to a relatively small sample size in this cohort. The median survival times were 55.4 and 32.2 months between predicted responders and non-responders among these UVA-55 patients.

### Clinical Utility of COXEN Predictors

Over ∼75% patients with ovarian cancer respond to the initial platinum-based chemotherapy. This leads to an important requirement for platinum-based chemotherapy biomarkers in ovarian cancer. First, it is impractical in treating patients to use the above mathematically-derived cutoff value, which considers the same weights both for false negatives and false positives. The number of false negatives, i.e. incorrectly predicted responders, is still not a small number at such a cutoff value (∼15% of all responders), which cannot be ethically used in clinical practice. Therefore, in order to examine clinical utility of our biomarkers, we defined the cutoff values by maximizing negative predictive value (NPV) on Dressman-119. We then independently evaluated these cutoffs on our UVA-55 cohort in a prospective manner, which showed significant improvement of NPV to 67% (10/15), 75% (6/8), and 71% (5/7) for the carboplatin, paclitaxel, and combination predictors, respectively (**Table 3**). We also found that these provided very high sensitivity 91%, 96%, and 93% with the three predictors. Therefore, using these cutoff values, one could identify ∼70% of the non-responders in advance to the primary platinum-based chemotherapy both on Dressman-119 and UVA-55 cohorts, maintaining >93% sensitivity (**Table 3**; Supplementary **Figure S2**).

We also investigated whether the chemotherapeutic response prediction could be improved by using other clinical parameters such as age, tumor stage, optimal debulking status, or race by a multivariate logistic regression analysis. We found that only the COXEN molecular predictor was a significant factor and other clinical variables were not significantly predictive of chemotherapy responses (Supplementary **Table S3**). Finally, we calculated the odds ratio of platinum-based chemotherapy response between predicted responders and predicted non-responders. The odds ratios were 7.16, 95% CI [1.315–38.912] for the Dressman-119 cohort and 4.51, 95% CI [1.013–20.096] for the UVA-55 cohort (Supplementary **Figure S3**). Therefore, the odds of chemotherapy response was >4.5 times higher for the predicted responder patients of the two diverse cohorts.

## Discussion

We have developed and independently validated multi-gene predictors for the two primary chemotherapeutic agents in ovarian cancer—carboplatin and paclitaxel. These molecular predictors significantly and consistently stratified the responder patients from the non-responders using two independent and distinctive patient cohorts in their clinical settings and tumor sample types. In particular, the identical predictors (and their pre-defined cutoff values) provided consistent prediction capability in both settings. We believe that this is quite encouraging, especially since they could be successfully applied with a high prediction performance both on the frozen tissue and the archived FFPE patient samples which may enable us to utilize these tests for a much wider retrospective validation and in clinical settings.

At the clinical optimal cutoff points (maximizing NPV), these predictors could identify >70% of non-responders to primary platinum-based chemotherapy who may be guided to choose different therapeutic options, potentially avoiding unnecessary toxicity. While this may provide a clinical utility in the primary chemotherapy to pre-select a small number of non-responder patients, it can be more useful in the second-line or subsequent chemotherapy selection for which the proportion of non-responders is significantly higher. However, we are well aware that these results may still not provide sufficient clinical utility to be used in the primary treatment setting. Instead, these single-drug predictors would be highly useful for patients receiving second-line and subsequent chemotherapeutic decisions for whom chemotherapeutic responses are much more heterogeneous (and response rates lower for each agent).

COXEN predictors showed statistically significant predictability simultaneously on the Dressman-119 and UVA-55 cohorts which were quite heterogeneous in their clinical settings and tumor tissue types, i.e. fresh frozen tumor vs. paraffin embedded. We also believe these were encouraging results. COXEN is best thought of as a screening tool for chemotherapy response, so we believe >90% sensitivity with ∼70% NPV seen in our independent testing demonstrates its general clinical utility.

Several additional points are worth mentioning. The cell lines for our *in vitro* ovarian cancer training were serous adenocarcinomas. The Dressman-119 cases were also reported to be serous adenocarcinomas. All patient-derived tumors were advanced stage (III–IV). We found no difference in the predictor performance with stratification by histology (data not shown). The COXEN approach is based on multiple filtering steps for discovering the most predictive biomarkers for an individual patients' therapeutic response. Its initial discovery starts from *in vitro* drug activity data of cancer cell lines to identify gene expression biomarkers only relevant to single drug activities, which is infeasible from human patient data since patient data are often confounded with their prognostic and other treatment factors. On the other hand, the majority of biomarkers initially discovered from cell line data are not similarly regulated and functioning in vivo. We believe this is why direct attempt to use the molecular observations from cell line data has been difficult to be translated to the clinical setting. We employed several biomarker filtering steps to avoid such pitfalls. One of our initial steps is to confirm whether candidate genes' expression changes observed between the sensitive and resistant cell lines (to the drug of interest) have been consistently shown in patients treated with the drug. For this we used a training patient set, Bonome-185, which is completely independent of our test sets. To obtain our final biomarkers, we used other filtering steps including the COXEN step which examines the concordant expression regulation networks among the chosen biomarkers. Note that all these filtering steps were performed independent of and prior to applying to our test sets.

Linear discriminant analysis (LDA) is one of the widely-used multivariate classification techniques in statistics. We have used other techniques such as SVM and logistic regression (data not shown), and found that the prediction performance was generally similar for the same data set. We here used LDA, taking advantage of its elegant prediction and inference capability, such as easy expansion to multi-classes and posterior probabilities of membership conditional on the observed data. Mechanisms of action and patient responses to carboplatin and taxol are believed to be independent, which is one of the reasons why this combination chemotherapy is widely used in ovarian cancer. However, there often exists a certain degree of correlation in patient response between different drugs. In our current study we assumed their independence in order to statistically derive the combination prediction scores from the two drugs' individual prediction scores. Despite this limitation, we found that COXEN prediction was generally more significantly predictive for the combination chemotherapy, which, we believe, partially justifies such an assumption.

Some limitations of this study should be noted. Our grouping of the validation cases into responders (CR) and non-responders (PR, SD, PD) were clinically justified because cases with CR had excellent long-term survival whereas those with PR, SD, PD patients had a variable outcome. However, most cases with PR had some degree of tumor response and therefore these cases were not strictly resistant to therapy even if their long-term benefit from chemotherapy remained uncertain. To address the impact of this dichotomization, we may need to correlate prediction scores with a residual cancer burden treated as a continuous response variable in a future study. Also, genomic data from patients treated with single drugs were not available for validation. The lack of data from patients with different single agent therapies also limits the ability to truly evaluate the regimen specificity of the cell-line derived signatures. Our current combination-drug predictor was mathematically derived from single-drug predictors, assuming independence of these drugs' activities. This combination-drug prediction modeling may be too naïve to capture the complexity of potential multi-drug interactions that can occur during treatment which may also need to be expanded based on a combination of drug activities on these cell lines.

Several intriguing questions remain: if platinum resistance could be predicted preoperatively—in the absence of any therapeutic advancement—would we change surgical management? Would we change adjuvant or neoadjuvant chemotherapy choices? Would we be able to individualize cancer care for women with ovarian cancer? We think these questions can only be explicitly answered in the setting of a prospective clinical trial. Nevertheless, the preliminary data presented here suggest that platinum resistant patients with ovarian cancer can be selectively guided based on our molecular assays.

All of our microarray and patient data have been submitted to the GEO web site and will be released upon publication.

## Supporting Information

Figure S1 **COXEN Biomarkers and Gene Networks for Carboplatin and Paclitaxel.** (A) IPA Network Analysis for Carboplatin COXEN Biomarkers. (B) Clustering heatmap analysis with major gene networks with x-axis responder (red) and non-responder (green) patients and y-axis with Cell cycle network (red), Cellular growth and prolife ration network (green), and Connective tissue development and function and other cancer gene network (yellow). (C) IPA Network Analysis for Paclitaxel COXEN Biomarkers.
(PDF)

Figure S2 **PPV and NPV Analysis for COXEN Predictors on UVA-55. Positive predictive value (PPV) and negative predictive value (NPV) were plotted by varying cutoff values for predicted responders for:** (A) Carboplatin Predictor, (B) Paclitaxel Predictor, (C) Combination Predictor.
(PDF)

Figure S3 **Odds Ratio of Platinum-based Chemotherapy Response between the Predictive Responders and Predictive Non-Responders.** The odds ratio of chemotherapy response was 4.5∼7.1 times favorable for the predictive responders both for Dressman-119 and UVA-55 patient sets, with 95% CI [1.315–38.912] and [1.013–20.096], respectively.
(PDF)

Table S1 **A:** Top gene networks among COXEN biomarkers. Information on COXEN biomarkers.
(DOC)

Table S2 Performance of COXEN Single and Combination Predictors at the Youden cutoff.
(DOC)

Table S3 A multivariate logistic regression analysis both with COXEN and other clinical variables on the UVA-55 cohort.
(DOC)

Text S1 (DOC)
