# Supplementary material for: Multi-Gene Expression Predictors of Single Drug Responses to Adjuvant Chemotherapy in Ovarian Carcinoma: Predicting Platinum Resistance
Source: PLoS One. 2012 Feb 10;7(2):e30550. doi: 10.1371/journal.pone.0030550 (PMC3277593; doi:10.1371/journal.pone.0030550)
Supplement: Figure S1 — COXEN Biomarkers and Gene Networks for Carboplatin and Paclitaxel. (A) IPA Network Analysis for Carboplatin COXEN Biomarkers. (B) Clustering heatmap analysis with major gene networks with x-axis responder (red) and non-responder (green) patients and y-axis with Cell cycle network (red), Cellular growth and prolife ration network (green), and Connective tissue development and function and other cancer gene network (yellow). (C) IPA Network Analysis for Paclitaxel COXEN Biomarkers. (PDF) [file pone.0030550.s001.pdf]

Figure S1A

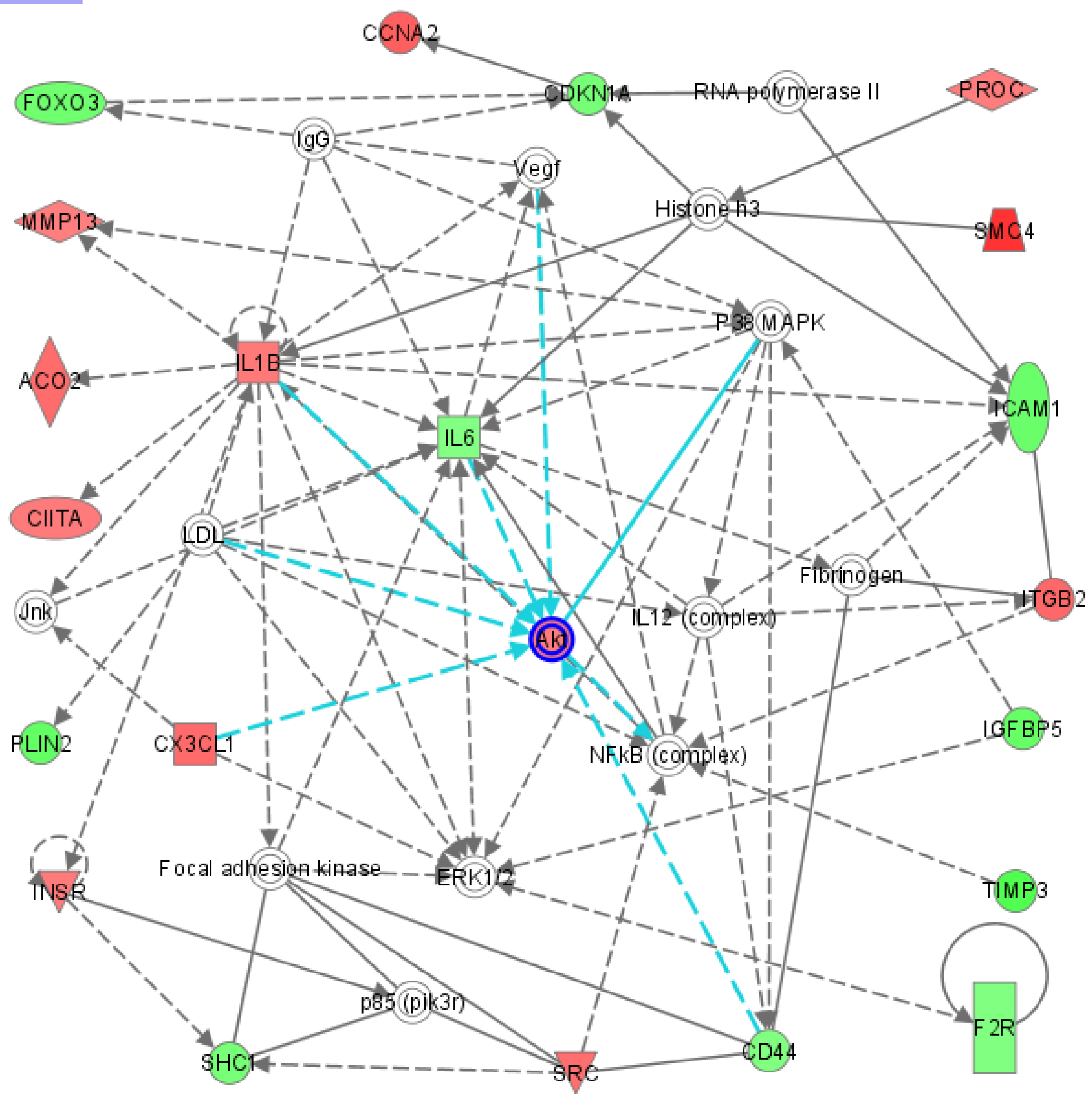
CCNA2
FOXO3
CDKN1A
RNA polymerase II
PROC
IgG
Vegf
MMP13
Histone h3
SMC4
P38 MAPK
IL1B
ACO2
IL6
ICAM1
CIITA
LDL
Fibrinogen
Jnk
IL12 (complex)
ITGB2
Akt
PLIN2
CX3CL1
NFkB (complex)
IGFBP5
Focal adhesion kinase
ERK1/2
INSR
TIMP3
p85 (pik3r)
F2R
SHC1
SRC
CD44

Figure S1B

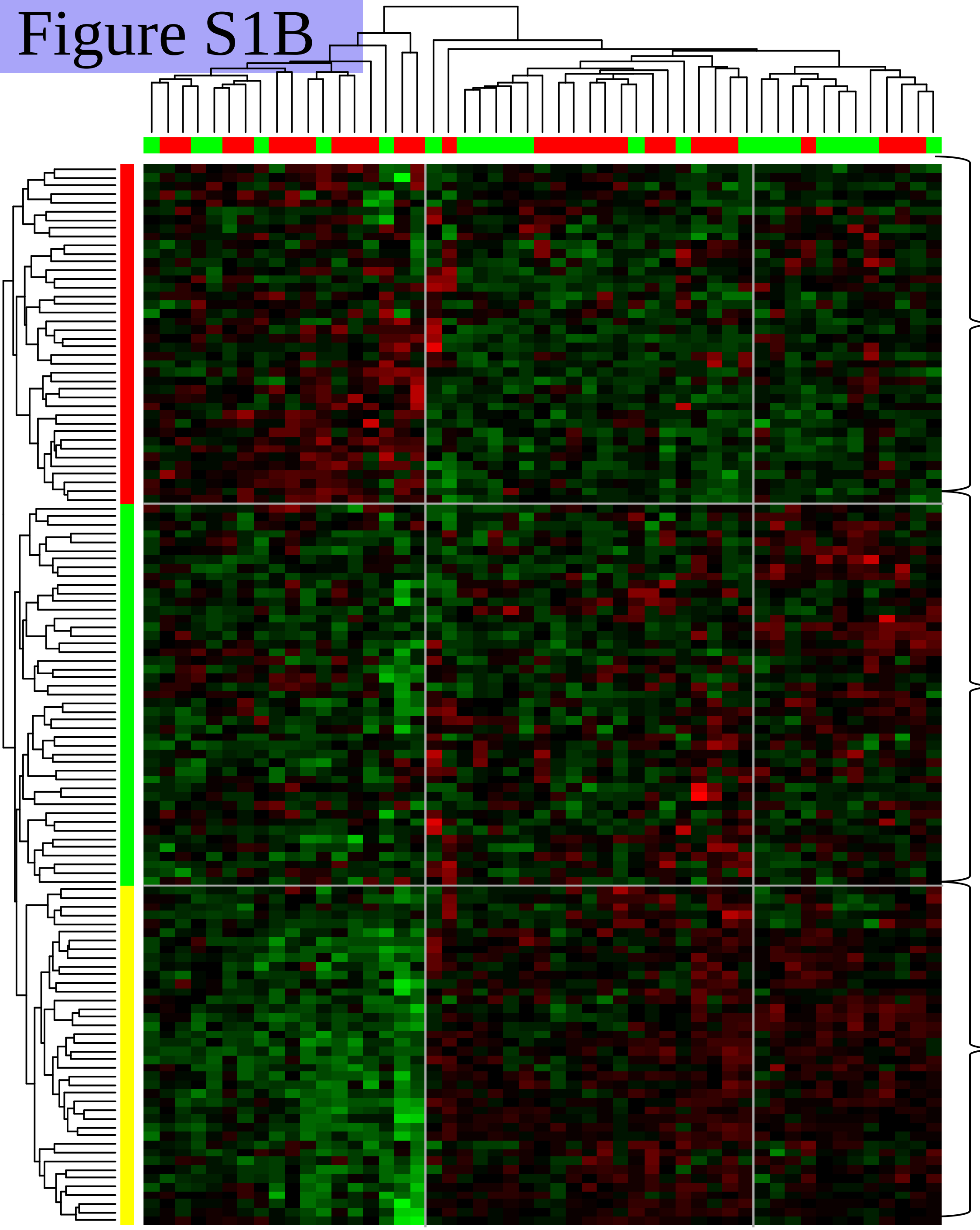

•Cell cycle, Hepatic System development and Function , Cancer

- MARCH3, NOC2L, TGFA, EGFR, ITGA3, P4HA2, ADCY9

•Cancer

- EGFR, GEMIN4, ITGA3, TGFA

•Cellular development, Cellular growth and prolife ration, Inflammatory disease

- SMAD3, TIMP2, VCAM1, EFNA5, CCNA1, PRPF4, TGM2, DUSP14, PEG10

•Hypersensitivity response

- ITGB3, VCAM1, TIMP2

•Cell cycle, Cellular growth and proliferation, Connective tissue development and function

- ACTN1, AMOTL2, DEGS1, ITGAV, MCM3, MYC, TPM1

• Cancer related genes

- MYC, ITGAV, FEN1, MCM3

Figure S1C

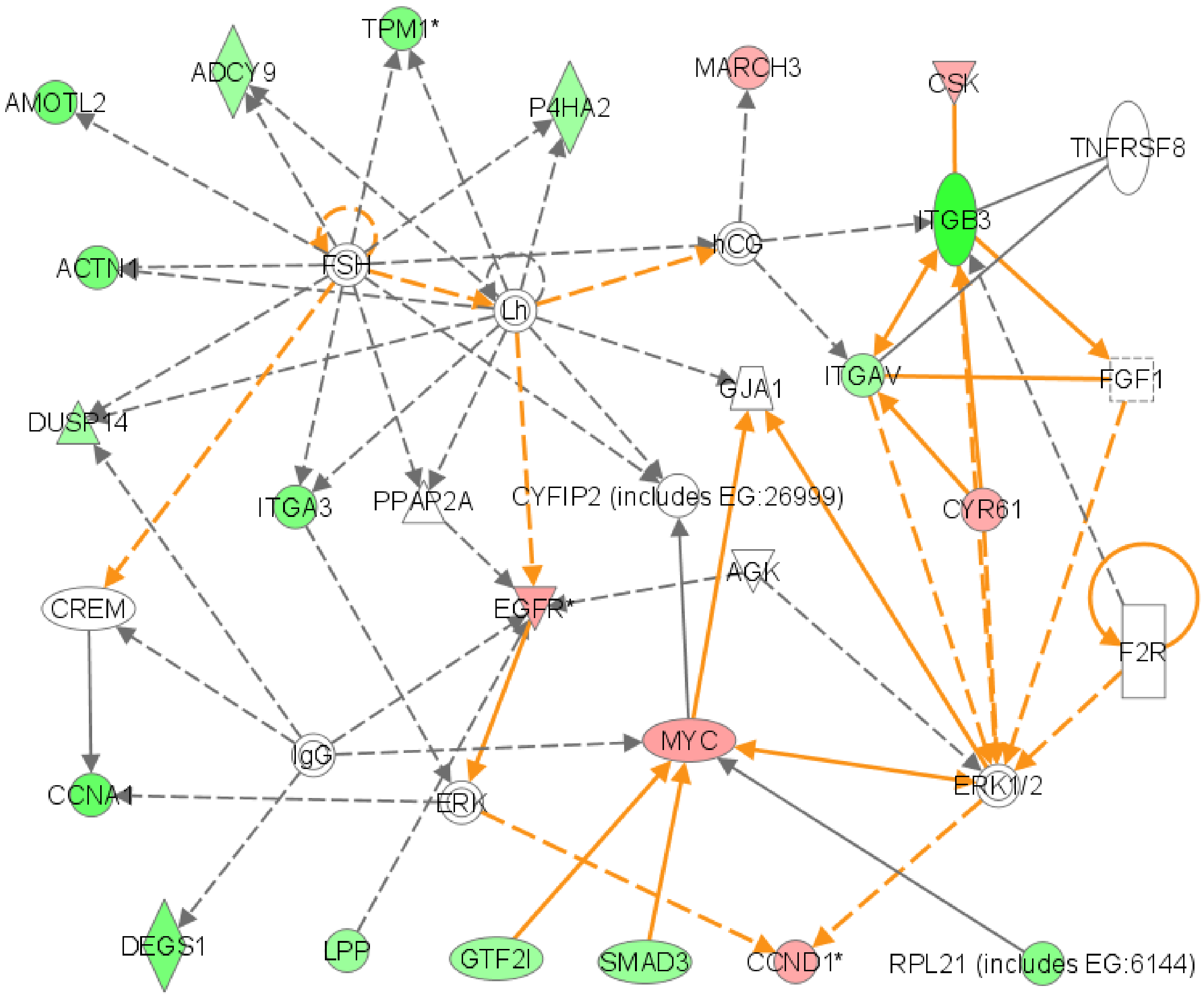
TPM1*
ADCY9
AMOTL2
P4HA2
MARCH3
CSK
TNFRSF8
ITGB3
hCG
FSH
ACTN1
Lh
ITGAV
FGF1
GJA1
DUSP14
ITGA3
PPAP2A
CYFIP2 (includes EG:26999)
CYR61
AGK
CREM
EGFR*
F2R
MYC
IgG
CCNA1
ERK
ERK1/2
DEGS1
LPP
GTF2I
SMAD3
CCND1*
RPL21 (includes EG:6144)
