# Supplementary figures and images for: Multi-Gene Expression Predictors of Single Drug Responses to Adjuvant Chemotherapy in Ovarian Carcinoma: Predicting Platinum Resistance
Source: PLoS One. 2012 Feb 10;7(2):e30550. doi: 10.1371/journal.pone.0030550 (PMC3277593; doi:10.1371/journal.pone.0030550)

Figure S2A

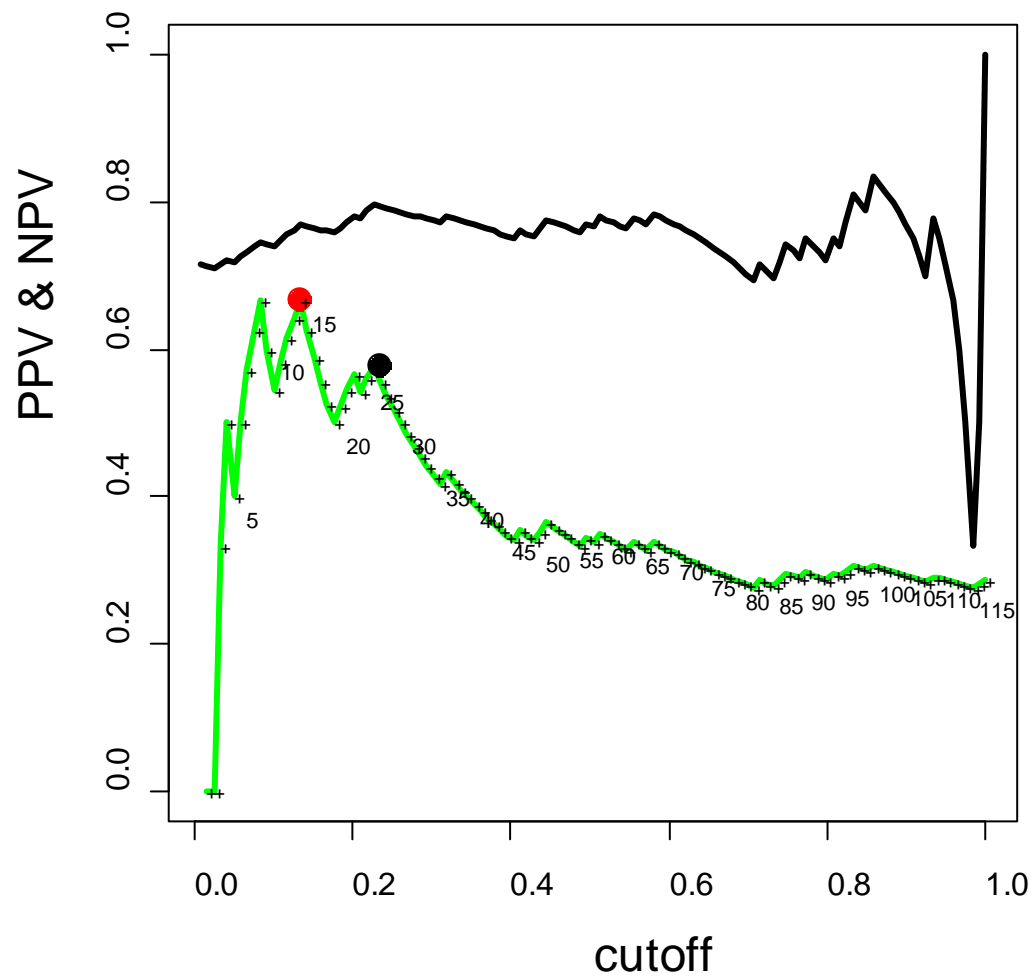

Figure S2B

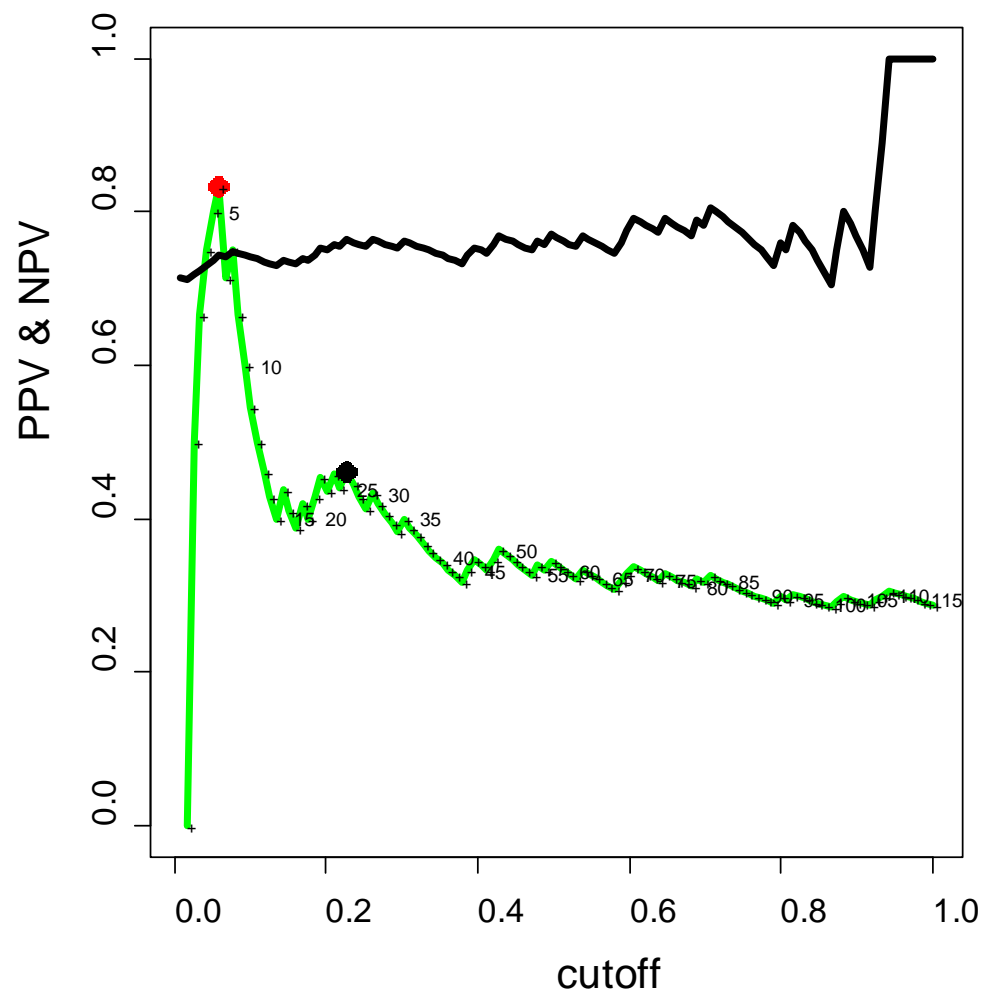

Figure S2C

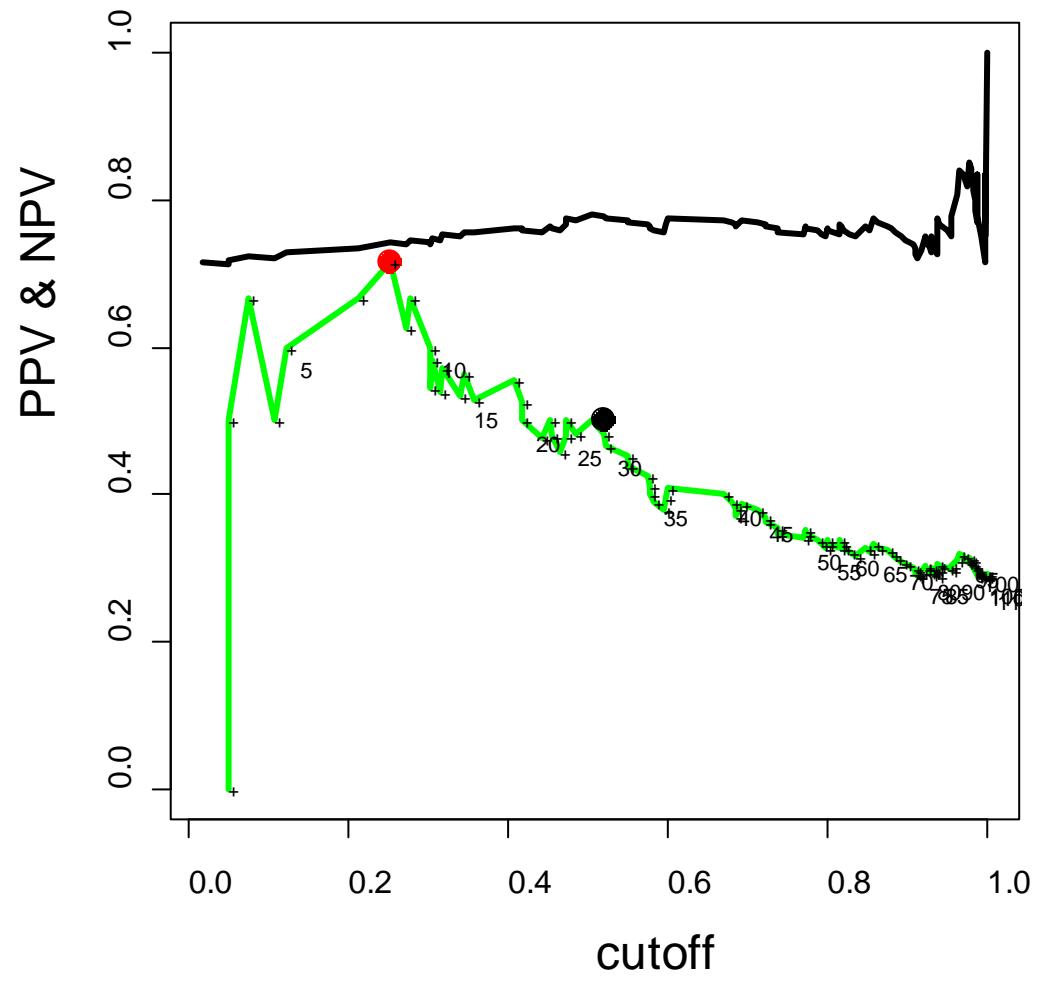

Supplement: Figure S2 — PPV and NPV Analysis for COXEN Predictors on UVA-55. Positive predictive value (PPV) and negative predictive value (NPV) were plotted by varying cutoff values for predicted responders for: (A) Carboplatin Predictor, (B) Paclitaxel Predictor, (C) Combination Predictor. (PDF) [file pone.0030550.s002.pdf]

**OR : Predicted Response  
for response**

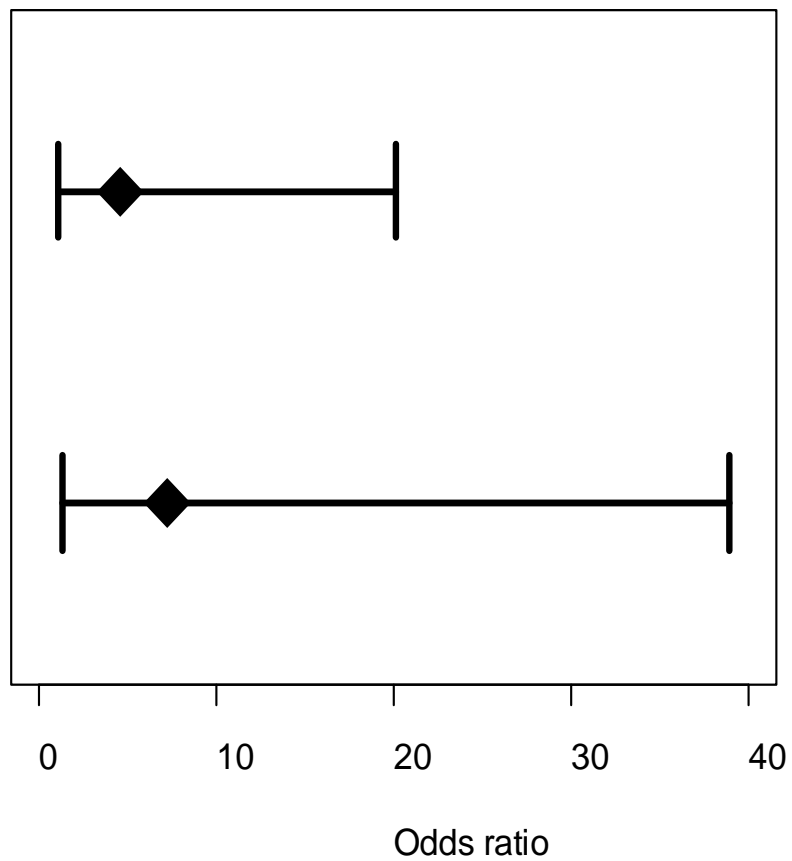

Supplement: Figure S3 — Odds Ratio of Platinum-based Chemotherapy Response between the Predictive Responders and Predictive Non-Responders. The odds ratio of chemotherapy response was 4.5∼7.1 times favorable for the predictive responders both for Dressman-119 and UVA-55 patient sets, with 95% CI [1.315–38.912] and [1.013–20.096], respectively. (PDF) [file pone.0030550.s003.pdf]
