# Supplementary material for: Multi-Gene Expression Predictors of Single Drug Responses to Adjuvant Chemotherapy in Ovarian Carcinoma: Predicting Platinum Resistance
Source: PLoS One. 2012 Feb 10;7(2):e30550. doi: 10.1371/journal.pone.0030550 (PMC3277593; doi:10.1371/journal.pone.0030550)
Supplement: Table S1 — A: Top gene networks among COXEN biomarkers. Information on COXEN biomarkers. (DOC) [file pone.0030550.s004.doc]

**Supplementary Table S1A: top gene networks among COXEN biomarkers**

| *Drug* | *Associated Network Functions* | *# genes* |
| --- | --- | --- |
| carboplatin | **Cell Cycle,** **Inflammatory Disease** | 29 |
|  | **Cell-To-Cell Signaling and Interactio**n, Hematological System Development and Function, Cellular Development | 20 |
|  | Organismal Functions, Cellular Movement, Carbohydrate Metabolism | 18 |
|  | Cell Cycle, **Cellular Movement**, Cellular Growth and Proliferation | 12 |
|  | Inflammatory Response, **Cellular Development,** Hematological System Development and Function | 9 |
| paclitaxel | Cellular Movement, Cell Death, Cardiovascular System Development and Function | 31 |
|  | DNA Replication, Recombination, and Repair, Cell Cycle, Cancer | 14 |
|  | Cell Morphology, Cellular Development, Cell-To-Cell Signaling and Interaction | 11 |
|  | Immunological Disease, Inflammatory Disease, Cell Death | 8 |
|  | Skeletal and Muscular System Development and Function, Tissue Morphology, Cellular Development | 2 |

[Table 2] Pathway analysis: Associated network functions

**Supplementary Table S1B: information on COXEN biomarkers**

**1) Biomarkers for Carboplatin**

|  | ***ID*** | ***Symbol*** | ***EntrezGeneName*** | ***Location*** |
| --- | --- | --- | --- | --- |
| 1 | 208288_at | ABCB11 | ATP-bindingcassette,sub-familyB(MDR/TAP),member11 | ExtracellularSpace |
| 2 | 200793_s_at | ACO2 | aconitase2,mitochondrial | Cytoplasm |
| 3 | 206840_at | AFM | afamin | ExtracellularSpace |
| 4 | 202834_at | AGT | angiotensinogen(serpinpeptidaseinhibitor,cladeA,member8) | ExtracellularSpace |
| 5 | 210517_s_at | AKAP12 | Akinase(PRKA)anchorprotein12 | Cytoplasm |
| 6 | 203910_at | ARHGAP29 | RhoGTPaseactivatingprotein29 | Cytoplasm |
| 7 | 211716_x_at | ARHGDIA | RhoGDPdissociationinhibitor(GDI)alpha | Cytoplasm |
| 8 | 214498_at | ASIP | agoutisignalingprotein,nonagoutihomolog(mouse) | ExtracellularSpace |
| 9 | 206030_at | ASPA | aspartoacylase(Canavandisease) | unknown |
| 10 | 201172_x_at | ATP6V0E1 | ATPase,H+transporting,lysosomal9kDa,V0subunite1 | Cytoplasm |
| 11 | 204624_at | ATP7B | ATPase,Cu++transporting,betapolypeptide | Cytoplasm |
| 12 | 202391_at | BASP1 | brainabundant,membraneattachedsignalprotein1 | PlasmaMembrane |
| 13 | 214068_at | BEAN | brainexpressed,associatedwithNedd4 | unknown |
| 14 | 202931_x_at | BIN1 | bridgingintegrator1 | Nucleus |
| 15 | 204832_s_at | BMPR1A | bonemorphogeneticproteinreceptor,typeIA | PlasmaMembrane |
| 16 | 207186_s_at | BPTF | bromodomainPHDfingertranscriptionfactor | Nucleus |
| 17 | 213346_at | C13ORF27 | chromosome13openreadingframe27 | unknown |
| 18 | 213143_at | C2ORF72 | chromosome2openreadingframe72 | unknown |
| 19 | 212848_s_at | C9ORF3 | chromosome9openreadingframe3 | Cytoplasm |
| 20 | 210404_x_at | CAMK2B | calcium/calmodulin-dependentproteinkinaseIIbeta | Cytoplasm |
| 21 | 207842_s_at | CASC3 | cancersusceptibilitycandidate3 | Nucleus |
| 22 | 206037_at | CCBL1 | cysteineconjugate-betalyase,cytoplasmic | Cytoplasm |
| 23 | 206887_at | CCBP2 | chemokinebindingprotein2 | PlasmaMembrane |
| 24 | 208094_s_at | CCDC130 | coiled-coildomaincontaining130 | unknown |
| 25 | 203418_at | CCNA2 | cyclinA2 | Nucleus |
| 26 | 204490_s_at | CD44 | CD44molecule(Indianbloodgroup) | PlasmaMembrane |
| 27 | 204996_s_at | CDK5R1 | cyclin-dependentkinase5,regulatorysubunit1(p35) | Nucleus |
| 28 | 202284_s_at | CDKN1A | cyclin-dependentkinaseinhibitor1A(p21,Cip1) | Nucleus |
| 29 | 207647_at | CDY1 | chromodomainprotein,Y-linked,1 | Nucleus |
| 30 | 213509_x_at | CES2(includesEG:8824) | carboxylesterase2(intestine,liver) | Cytoplasm |
| 31 | 203854_at | CFI | complementfactorI | ExtracellularSpace |
| 32 | 209763_at | CHRDL1 | chordin-like1 | ExtracellularSpace |
| 33 | 207274_at | CHRNE | cholinergicreceptor,nicotinic,epsilon | PlasmaMembrane |
| 34 | 201953_at | CIB1 | calciumandintegrinbinding1(calmyrin) | Nucleus |
| 35 | 205101_at | CIITA | classII,majorhistocompatibilitycomplex,transactivator | Nucleus |
| 36 | 207047_s_at | CLCNKB | chloridechannelKb | PlasmaMembrane |
| 37 | 205723_at | CNTFR | ciliaryneurotrophicfactorreceptor | PlasmaMembrane |
| 38 | 214602_at | COL4A4(includesEG:1286) | collagen,typeIV,alpha4 | ExtracellularSpace |
| 39 | 204136_at | COL7A1 | collagen,typeVII,alpha1 | ExtracellularSpace |
| 40 | 205489_at | CRYM | crystallin,mu | Cytoplasm |
| 41 | 200621_at | CSRP1 | cysteineandglycine-richprotein1 | Nucleus |
| 42 | 203687_at | CX3CL1 | chemokine(C-X3-Cmotif)ligand1 | ExtracellularSpace |
| 43 | 209975_at | CYP2E1 | cytochromeP450,family2,subfamilyE,polypeptide1 | Cytoplasm |
| 44 | 205939_at | CYP3A7 | cytochromeP450,family3,subfamilyA,polypeptide7 | Cytoplasm |
| 45 | 201095_at | DAP | death-associatedprotein | Cytoplasm |
| 46 | 213865_at | DCBLD2 | discoidin,CUBandLCCLdomaincontaining2 | PlasmaMembrane |
| 47 | 210811_s_at | DDX49 | DEAD(Asp-Glu-Ala-Asp)boxpolypeptide49 | Nucleus |
| 48 | 201386_s_at | DHX15 | DEAH(Asp-Glu-Ala-His)boxpolypeptide15 | Nucleus |
| 49 | 216870_x_at | DLEU2 | deletedinlymphocyticleukemia2(non-proteincoding) | unknown |
| 50 | 205554_s_at | DNASE1L3 | deoxyribonucleaseI-like3 | Nucleus |
| 51 | 213482_at | DOCK3 | dedicatorofcytokinesis3 | Cytoplasm |
| 52 | 202673_at | DPM1 | dolichyl-phosphatemannosyltransferasepolypeptide1,catalyticsubunit | Cytoplasm |
| 53 | 200762_at | DPYSL2 | dihydropyrimidinase-like2 | Cytoplasm |
| 54 | 208215_x_at | DRD4 | dopaminereceptorD4 | PlasmaMembrane |
| 55 | 208201_at | DUX3 | doublehomeobox,3 | Nucleus |
| 56 | 201999_s_at | DYNLT1 | dynein,lightchain,Tctex-type1 | Cytoplasm |
| 57 | 210151_s_at | DYRK3 | dual-specificitytyrosine-(Y)-phosphorylationregulatedkinase3 | Nucleus |
| 58 | 201510_at | ELF3 | E74-likefactor3(etsdomaintranscriptionfactor,epithelial-specific) | Nucleus |
| 59 | 220624_s_at | ELF5 | E74-likefactor5(etsdomaintranscriptionfactor) | Nucleus |
| 60 | 205162_at | ERCC8 | excisionrepaircross-complementingrodentrepairdeficiency,complementationgroup8 | Nucleus |
| 61 | 208858_s_at | ESYT1 | extendedsynaptotagmin-likeprotein1 | unknown |
| 62 | 206501_x_at | ETV1 | etsvariant1 | Nucleus |
| 63 | 209692_at | EYA2 | eyesabsenthomolog2(Drosophila) | Nucleus |
| 64 | 203989_x_at | F2R | coagulationfactorII(thrombin)receptor | PlasmaMembrane |
| 65 | 205892_s_at | FABP1 | fattyacidbindingprotein1,liver | Cytoplasm |
| 66 | 205029_s_at | FABP7 | fattyacidbindingprotein7,brain | Cytoplasm |
| 67 | 204007_at | FCGR3B | FcfragmentofIgG,lowaffinityIIIb,receptor(CD16b) | PlasmaMembrane |
| 68 | 204767_s_at | FEN1 | flapstructure-specificendonuclease1 | Nucleus |
| 69 | 205588_s_at | FGFR1OP(includesEG:11116) | FGFR1oncogenepartner | Cytoplasm |
| 70 | 208228_s_at | FGFR2 | fibroblastgrowthfactorreceptor2 | PlasmaMembrane |
| 71 | 202949_s_at | FHL2 | fourandahalfLIMdomains2 | Nucleus |
| 72 | 212024_x_at | FLII | flightlessIhomolog(Drosophila) | Nucleus |
| 73 | 207876_s_at | FLNC | filaminC,gamma | Cytoplasm |
| 74 | 201350_at | FLOT2 | flotillin2 | PlasmaMembrane |
| 75 | 205666_at | FMO1 | flavincontainingmonooxygenase1 | Cytoplasm |
| 76 | 204131_s_at | FOXO3 | forkheadboxO3 | Nucleus |
| 77 | 215380_s_at | GGCT | gamma-glutamylcyclotransferase | unknown |
| 78 | 207131_x_at | GGT1 | gamma-glutamyltransferase1 | Cytoplasm |
| 79 | 204762_s_at | GNAO1 | guaninenucleotidebindingprotein(Gprotein),alphaactivatingactivitypolypeptideO | PlasmaMembrane |
| 80 | 214227_at | GNG7 | guaninenucleotidebindingprotein(Gprotein),gamma7 | PlasmaMembrane |
| 81 | 210328_at | GNMT | glycineN-methyltransferase | Cytoplasm |
| 82 | 208465_at | GRM2 | glutamatereceptor,metabotropic2 | PlasmaMembrane |
| 83 | 203815_at | GSTT1 | glutathioneS-transferasetheta1 | Cytoplasm |
| 84 | 203817_at | GUCY1B3 | guanylatecyclase1,soluble,beta3 | Cytoplasm |
| 85 | 211275_s_at | GYG1 | glycogenin1 | Cytoplasm |
| 86 | 210164_at | GZMB | granzymeB(granzyme2,cytotoxicT-lymphocyte-associatedserineesterase1) | Cytoplasm |
| 87 | 206666_at | GZMK | granzymeK(granzyme3;tryptaseII) | Cytoplasm |
| 88 | 211222_s_at | HAP1 | huntingtin-associatedprotein1 | Cytoplasm |
| 89 | 204848_x_at | HBG2 | hemoglobin,gammaG | Cytoplasm |
| 90 | 202300_at | HBXIP | hepatitisBvirusxinteractingprotein | Cytoplasm |
| 91 | 201209_at | HDAC1 | histonedeacetylase1 | Nucleus |
| 92 | 201833_at | HDAC2 | histonedeacetylase2 | Nucleus |
| 93 | 209558_s_at | HIP1R | huntingtininteractingprotein1related | Cytoplasm |
| 94 | 213932_x_at | HLA-A | majorhistocompatibilitycomplex,classI,A | PlasmaMembrane |
| 95 | 200944_s_at | HMGN1 | high-mobilitygroupnucleosomebindingdomain1 | Nucleus |
| 96 | 205466_s_at | HS3ST1 | heparansulfate(glucosamine)3-O-sulfotransferase1 | Cytoplasm |
| 97 | 202638_s_at | ICAM1 | intercellularadhesionmolecule1 | PlasmaMembrane |
| 98 | 203153_at | IFIT1 | interferon-inducedproteinwithtetratricopeptiderepeats1 | Cytoplasm |
| 99 | 202718_at | IGFBP2 | insulin-likegrowthfactorbindingprotein2,36kDa | ExtracellularSpace |
| 100 | 211959_at | IGFBP5 | insulin-likegrowthfactorbindingprotein5 | ExtracellularSpace |
| 101 | 215420_at | IHH | Indianhedgehoghomolog(Drosophila) | ExtracellularSpace |
| 102 | 205067_at | IL1B | interleukin1,beta | ExtracellularSpace |
| 103 | 205207_at | IL6 | interleukin6(interferon,beta2) | ExtracellularSpace |
| 104 | 205070_at | ING3 | inhibitorofgrowthfamily,member3 | Nucleus |
| 105 | 207851_s_at | INSR | insulinreceptor | PlasmaMembrane |
| 106 | 203941_at | INTS9 | integratorcomplexsubunit9 | unknown |
| 107 | 202803_s_at | ITGB2 | integrin,beta2(complementcomponent3receptor3and4subunit) | PlasmaMembrane |
| 108 | 208083_s_at | ITGB6 | integrin,beta6 | PlasmaMembrane |
| 109 | 202746_at | ITM2A | integralmembraneprotein2A | PlasmaMembrane |
| 110 | 212733_at | KIAA0226 | KIAA0226 | unknown |
| 111 | 209661_at | KIFC3 | kinesinfamilymemberC3 | Cytoplasm |
| 112 | 214471_x_at | LHB | luteinizinghormonebetapolypeptide | ExtracellularSpace |
| 113 | 206230_at | LHX1 | LIMhomeobox1 | Nucleus |
| 114 | 204424_s_at | LMO3 | LIMdomainonly3(rhombotin-like2) | Nucleus |
| 115 | 211353_at | LRIT1 | leucine-richrepeat,immunoglobulin-likeandtransmembranedomains1 | Cytoplasm |
| 116 | 214460_at | LSAMP | limbicsystem-associatedmembraneprotein | PlasmaMembrane |
| 117 | 202655_at | MANF | mesencephalicastrocyte-derivedneurotrophicfactor | ExtracellularSpace |
| 118 | 213178_s_at | MAPK8IP3 | mitogen-activatedproteinkinase8interactingprotein3 | Cytoplasm |
| 119 | 209035_at | MDK | midkine(neuritegrowth-promotingfactor2) | ExtracellularSpace |
| 120 | 205124_at | MEF2B | myocyteenhancerfactor2B | Nucleus |
| 121 | 205959_at | MMP13 | matrixmetallopeptidase13(collagenase3) | ExtracellularSpace |
| 122 | 207012_at | MMP16 | matrixmetallopeptidase16(membrane-inserted) | ExtracellularSpace |
| 123 | 205106_at | MTCP1 | matureT-cellproliferation1 | Cytoplasm |
| 124 | 202364_at | MXI1 | MAXinteractor1 | Nucleus |
| 125 | 214087_s_at | MYBPC1 | myosinbindingproteinC,slowtype | Cytoplasm |
| 126 | 203360_s_at | MYCBP | c-mycbindingprotein | Nucleus |
| 127 | 205610_at | MYOM1 | myomesin1,185kDa | Cytoplasm |
| 128 | 212843_at | NCAM1 | neuralcelladhesionmolecule1 | PlasmaMembrane |
| 129 | 215205_x_at | NCOR2 | nuclearreceptorco-repressor2 | Nucleus |
| 130 | 202607_at | NDST1 | N-deacetylase/N-sulfotransferase(heparanglucosaminyl)1 | Cytoplasm |
| 131 | 204702_s_at | NFE2L3 | nuclearfactor(erythroid-derived2)-like3 | Nucleus |
| 132 | 207380_x_at | NOX1 | NADPHoxidase1 | Cytoplasm |
| 133 | 213040_s_at | NPTXR | neuronalpentraxinreceptor | PlasmaMembrane |
| 134 | 209483_s_at | NSL1 | NSL1,MINDkinetochorecomplexcomponent,homolog(S.cerevisiae) | Nucleus |
| 135 | 210797_s_at | OASL | 2'-5'-oligoadenylatesynthetase-like | unknown |
| 136 | 204088_at | P2RX4 | purinergicreceptorP2X,ligand-gatedionchannel,4 | PlasmaMembrane |
| 137 | 212259_s_at | PBXIP1 | pre-B-cellleukemiahomeoboxinteractingprotein1 | Nucleus |
| 138 | 205689_at | PCNXL2 | pecanex-like2(Drosophila) | unknown |
| 139 | 205463_s_at | PDGFA | platelet-derivedgrowthfactoralphapolypeptide | ExtracellularSpace |
| 140 | 213302_at | PFAS | phosphoribosylformylglycinamidinesynthase | Cytoplasm |
| 141 | 202328_s_at | PKD1 | polycystickidneydisease1(autosomaldominant) | PlasmaMembrane |
| 142 | 209532_at | PLAA | phospholipaseA2-activatingprotein | Cytoplasm |
| 143 | 203471_s_at | PLEK | pleckstrin | Cytoplasm |
| 144 | 209122_at | PLIN2 | perilipin2 | PlasmaMembrane |
| 145 | 205190_at | PLS1 | plastin1 | Cytoplasm |
| 146 | 210139_s_at | PMP22 | peripheralmyelinprotein22 | PlasmaMembrane |
| 147 | 207448_at | POFUT2 | proteinO-fucosyltransferase2 | Cytoplasm |
| 148 | 205478_at | PPP1R1A | proteinphosphatase1,regulatory(inhibitor)subunit1A | Cytoplasm |
| 149 | 213774_s_at | PPP1R2 | proteinphosphatase1,regulatory(inhibitor)subunit 2 | Cytoplasm |
| 150 | 211084_x_at | PRKD3 | proteinkinaseD3 | unknown |
| 151 | 206259_at | PROC | proteinC(inactivatorofcoagulationfactorsVaandVIIIa) | ExtracellularSpace |
| 152 | 205847_at | PRSS22 | protease,serine,22 | ExtracellularSpace |
| 153 | 204279_at | PSMB9 | proteasome(prosome,macropain)subunit,betatype,9(largemultifunctionalpeptidase2) | Cytoplasm |
| 154 | 208617_s_at | PTP4A2 | proteintyrosinephosphatasetypeIVA,member2 | PlasmaMembrane |
| 155 | 201140_s_at | RAB5C | RAB5C,memberRASoncogenefamily | Cytoplasm |
| 156 | 206103_at | RAC3 | ras-relatedC3botulinumtoxinsubstrate3(rhofamily,smallGTPbindingproteinRac3) | Cytoplasm |
| 157 | 212127_at | RANGAP1 | RanGTPaseactivatingprotein1 | Cytoplasm |
| 158 | 215683_at | RBFA | Ribosome binding factor A (putative) | Cytoplasm |
| 159 | 202296_s_at | RER1 | RER1retentioninendoplasmicreticulum1homolog(S.cerevisiae) | Cytoplasm |
| 160 | 205578_at | ROR2 | receptortyrosinekinase-likeorphanreceptor2 | PlasmaMembrane |
| 161 | 200888_s_at | RPL23 | ribosomalproteinL23 | Cytoplasm |
| 162 | 213801_x_at | RPSA | ribosomalproteinSA | PlasmaMembrane |
| 163 | 200872_at | S100A10 | S100calciumbindingproteinA10 | Cytoplasm |
| 164 | 206995_x_at | SCARF1 | scavengerreceptorclassF,member1 | PlasmaMembrane |
| 165 | 204035_at | SCG2 | secretograninII(chromograninC) | ExtracellularSpace |
| 166 | 204541_at | SEC14L2 | SEC14-like2(S.cerevisiae) | Cytoplasm |
| 167 | 203871_at | SENP3 | SUMO1/sentrin/SMT3specificpeptidase3 | Nucleus |
| 168 | 205576_at | SERPIND1 | serpinpeptidaseinhibitor,cladeD(heparincofactor),member1 | ExtracellularSpace |
| 169 | 202283_at | SERPINF1 | serpinpeptidaseinhibitor,cladeF(alpha-2antiplasmin,pigmentepitheliumderivedfactor),member1 | ExtracellularSpace |
| 170 | 205121_at | SGCB | sarcoglycan,beta(43kDadystrophin-associatedglycoprotein) | PlasmaMembrane |
| 171 | 209402_s_at | SLC12A4 | solutecarrierfamily12(potassium/chloridetransporters),member4 | PlasmaMembrane |
| 172 | 202800_at | SLC1A3 | solutecarrierfamily1(glialhighaffinityglutamatetransporter),member3 | PlasmaMembrane |
| 173 | 201249_at | SLC2A1 | solutecarrierfamily2(facilitatedglucosetransporter),member1 | PlasmaMembrane |
| 174 | 211123_at | SLC5A5 | solutecarrierfamily5(sodiumiodidesymporter),member5 | PlasmaMembrane |
| 175 | 206836_at | SLC6A3 | solutecarrierfamily6(neurotransmittertransporter,dopamine),member3 | PlasmaMembrane |
| 176 | 201663_s_at | SMC4 | structuralmaintenanceofchromosomes4 | Nucleus |
| 177 | 210057_at | SMG1 | SMG1homolog,phosphatidylinositol3-kinase-relatedkinase(C.elegans) | Cytoplasm |
| 178 | 205573_s_at | SNX7 | sortingnexin7 | unknown |
| 179 | 210677_at | SOAT2 | sterolO-acyltransferase2 | Cytoplasm |
| 180 | 208468_at | SOX21 | SRY(sexdeterminingregionY)-box21 | Nucleus |
| 181 | 205155_s_at | SPTBN2 | spectrin,beta,non-erythrocytic2 | Cytoplasm |
| 182 | 221284_s_at | SRC | v-srcsarcoma(Schmidt-RuppinA-2)viraloncogenehomolog(avian) | Cytoplasm |
| 183 | 202440_s_at | ST5 | suppressionoftumorigenicity5 | unknown |
| 184 | 212111_at | STX12 | syntaxin12 | PlasmaMembrane |
| 185 | 208207_at | STX1B | syntaxin1B | PlasmaMembrane |
| 186 | 214304_x_at | SYNM | synemin,intermediatefilamentprotein | Cytoplasm |
| 187 | 203999_at | SYT1 | synaptotagminI | Cytoplasm |
| 188 | 206409_at | TIAM1 | T-celllymphomainvasionandmetastasis1 | Cytoplasm |
| 189 | 213135_at | TIAM1 | T-celllymphomainvasionandmetastasis1 | Cytoplasm |
| 190 | 203167_at | TIMP2 | TIMPmetallopeptidaseinhibitor2 | ExtracellularSpace |
| 191 | 201149_s_at | TIMP3 | TIMPmetallopeptidaseinhibitor3 | ExtracellularSpace |
| 192 | 206179_s_at | TPPP | tubulinpolymerizationpromotingprotein | Cytoplasm |
| 193 | 215047_at | TRIM58 | tripartitemotif-containing58 | unknown |
| 194 | 212664_at | TUBB4 | tubulin,beta4 | Cytoplasm |
| 195 | 211915_s_at | TUBB4Q | tubulin,betapolypeptide4,memberQ | unknown |
| 196 | 206716_at | UMOD | uromodulin | ExtracellularSpace |
| 197 | 214624_at | UPK1A | uroplakin1A | PlasmaMembrane |
| 198 | 210064_s_at | UPK1B | uroplakin1B | PlasmaMembrane |
| 199 | 205139_s_at | UST | uronyl-2-sulfotransferase | Cytoplasm |
| 200 | 212156_at | VPS39 | vacuolarproteinsorting39homolog(S.cerevisiae) | Cytoplasm |
| 201 | 209452_s_at | VTI1B | vesicletransportthroughinteractionwitht-SNAREshomolog1B(yeast) | PlasmaMembrane |
| 202 | 219077_s_at | WWOX | WWdomaincontainingoxidoreductase | Cytoplasm |
| 203 | 213725_x_at | XYLT1 | xylosyltransferaseI | Cytoplasm |
| 204 | 214713_at | YLPM1 | YLPmotifcontaining1 | Nucleus |
| 205 | 210996_s_at | YWHAE | tyrosine3-monooxygenase/tryptophan5-monooxygenaseactivationprotein,epsilonpolypeptide | Cytoplasm |
| 206 | 203603_s_at | ZEB2 | zincfingerE-boxbindinghomeobox2 | Nucleus |
| 207 | 202136_at | ZMYND11 | zincfinger,MYNDdomaincontaining11 | Nucleus |
| 208 | 206928_at | ZNF124 | zincfingerprotein124 | Nucleus |
| 209 | 206314_at | ZNF167 | zincfingerprotein167 | Nucleus |
| 210 | 207781_s_at | ZNF711 | zincfingerprotein711 | Nucleus |
| 211 | 216650_at | RPL29P17 | Ribosomal protein L29 pseudogene 17 |  |
| 212 | 215697_at | AF052172 | Homosapiens clone 24617 |  |
| 213 | 216659_at | LOC1720 |  |  |
| 214 | 207881_at | AF050199 | Homosapiens putative peroxisome microbody protein 175.1 |  |
| 215 | 217048_at | Y09846 |  |  |

**2) Biomarkers for Paclitaxel**

|  | ***ID*** | ***Symbol*** | ***EntrezGeneName*** | ***Location*** |
| --- | --- | --- | --- | --- |
| 1 | 214033_at | ABCC6 | ATP-bindingcassette,sub-familyC(CFTR/MRP),member6 | PlasmaMembrane |
| 2 | 208636_at | ACTN1 | actinin,alpha1 | Cytoplasm |
| 3 | 204497_at | ADCY9 | adenylatecyclase9 | PlasmaMembrane |
| 4 | 211986_at | AHNAK | AHNAKnucleoprotein | Nucleus |
| 5 | 203180_at | ALDH1A3 | aldehydedehydrogenase1family,memberA3 | Cytoplasm |
| 6 | 203002_at | AMOTL2 | angiomotinlike2 | PlasmaMembrane |
| 7 | 203910_at | ARHGAP29 | RhoGTPaseactivatingprotein29 | Cytoplasm |
| 8 | 206414_s_at | ASAP2 | ArfGAPwithSH3domain,ankyrinrepeatandPHdomain2 | Nucleus |
| 9 | 209394_at | ASMTL(includesEG:8623) | acetylserotoninO-methyltransferase-like | unknown |
| 10 | 205047_s_at | ASNS | asparaginesynthetase | unknown |
| 11 | 208898_at | ATP6V1D | ATPase,H+transporting,lysosomal34kDa,V1subunitD | Cytoplasm |
| 12 | 213429_at | BICC1 | Bicaudal C homolog 1 | unknown |
| 13 | 203773_x_at | BLVRA | biliverdinreductaseA | Cytoplasm |
| 14 | 212923_s_at | C6ORF145 | chromosome6openreadingframe145 | unknown |
| 15 | 205899_at | CCNA1 | cyclinA1 | Nucleus |
| 16 | 208712_at | CCND1 | cyclinD1 | Nucleus |
| 17 | 208711_s_at | CCND1 | cyclinD1 | Nucleus |
| 18 | 209583_s_at | CD200 | CD200molecule | PlasmaMembrane |
| 19 | 200663_at | CD63 | CD63molecule | PlasmaMembrane |
| 20 | 203377_s_at | CDC40 | celldivisioncycle40homolog(S.cerevisiae) | Nucleus |
| 21 | 209834_at | CHST3 | carbohydrate(chondroitin6)sulfotransferase3 | Cytoplasm |
| 22 | 202110_at | COX7B | cytochromecoxidasesubunitVIIb | Cytoplasm |
| 23 | 206256_at | CPN1 | carboxypeptidaseN,polypeptide1 | ExtracellularSpace |
| 24 | 202329_at | CSK | c-srctyrosinekinase | Cytoplasm |
| 25 | 204459_at | CSTF2(includesEG:1478) | cleavagestimulationfactor,3'pre-RNA,subunit2,64kDa | Nucleus |
| 26 | 202295_s_at | CTSH | cathepsinH | Cytoplasm |
| 27 | 201289_at | CYR61 | cysteine-rich,angiogenicinducer,61 | ExtracellularSpace |
| 28 | 210811_s_at | DDX49 | DEAD(Asp-Glu-Ala-Asp)boxpolypeptide49 | Nucleus |
| 29 | 209250_at | DEGS1 | degenerativespermatocytehomolog1,lipiddesaturase(Drosophila) | PlasmaMembrane |
| 30 | 203695_s_at | DFNA5 | deafness,autosomaldominant5 | unknown |
| 31 | 207556_s_at | DGKZ | diacylglycerolkinase,zeta104kDa | Cytoplasm |
| 32 | 212504_at | DIP2C | DIP2disco-interactingprotein2homologC(Drosophila) | unknown |
| 33 | 201479_at | DKC1 | dyskeratosiscongenita1,dyskerin | Nucleus |
| 34 | 203367_at | DUSP14 | dualspecificityphosphatase14 | unknown |
| 35 | 204794_at | DUSP2 | dualspecificityphosphatase2 | Nucleus |
| 36 | 214036_at | EFNA5 | ephrin-A5 | PlasmaMembrane |
| 37 | 201983_s_at | EGFR | epidermalgrowthfactorreceptor(erythroblasticleukemiaviral(v-erb-b)oncogenehomolog,avian) | PlasmaMembrane |
| 38 | 201984_s_at | EGFR | epidermalgrowthfactorreceptor(erythroblasticleukemiaviral(v-erb-b)oncogenehomolog,avian) | PlasmaMembrane |
| 39 | 204643_s_at | ENOX2 | ecto-NOXdisulfide-thiolexchanger2 | PlasmaMembrane |
| 40 | 208092_s_at | FAM49A | familywithsequencesimilarity49,memberA | unknown |
| 41 | 214436_at | FBXL2 | F-boxandleucine-richrepeatprotein2 | Cytoplasm |
| 42 | 204767_s_at | FEN1 | flapstructure-specificendonuclease1 | Nucleus |
| 43 | 206857_s_at | FKBP1B | FK506bindingprotein1B,12.6kDa | Cytoplasm |
| 44 | 203091_at | FUBP1 | farupstreamelement(FUSE)bindingprotein1 | Nucleus |
| 45 | 205527_s_at | GEMIN4 | gem(nuclearorganelle)associatedprotein4 | Nucleus |
| 46 | 205696_s_at | GFRA1 | GDNFfamilyreceptoralpha1 | PlasmaMembrane |
| 47 | 204115_at | GNG11 | guaninenucleotidebindingprotein(Gprotein),gamma11 | PlasmaMembrane |
| 48 | 204137_at | GPR137B | Gprotein-coupledreceptor137B | PlasmaMembrane |
| 49 | 202554_s_at | GSTM3(includesEG:2947) | glutathioneS-transferasemu3(brain) | Cytoplasm |
| 50 | 205439_at | GSTT2 | glutathioneS-transferasetheta2 | Cytoplasm |
| 51 | 210892_s_at | GTF2I | generaltranscriptionfactorIIi | Nucleus |
| 52 | 204805_s_at | H1FX | H1histonefamily,memberX | Nucleus |
| 53 | 208668_x_at | HMGN2 | high-mobilitygroupnucleosomalbindingdomain2 | Nucleus |
| 54 | 205453_at | HOXB2 | homeoboxB2 | Nucleus |
| 55 | 201162_at | IGFBP7 | insulin-likegrowthfactorbindingprotein7 | ExtracellularSpace |
| 56 | 201474_s_at | ITGA3 | integrin,alpha3(antigenCD49C,alpha3subunitofVLA-3receptor) | PlasmaMembrane |
| 57 | 202351_at | ITGAV | integrin,alphaV(vitronectinreceptor,alphapolypeptide,antigenCD51) | PlasmaMembrane |
| 58 | 204627_s_at | ITGB3 | integrin,beta3(plateletglycoproteinIIIa,antigenCD61) | PlasmaMembrane |
| 59 | 201505_at | LAMB1 | laminin,beta1 | ExtracellularSpace |
| 60 | 202378_s_at | LEPROT | leptinreceptoroverlappingtranscript | PlasmaMembrane |
| 61 | 203276_at | LMNB1 | laminB1 | Nucleus |
| 62 | 210178_x_at | LOC100505793 | Serine/arginine-rich splicing factor 10 | Nucleus |
| 63 | 202822_at | LPP | LIMdomaincontainingpreferredtranslocationpartnerinlipoma | Nucleus |
| 64 | 203362_s_at | MAD2L1 | MAD2mitoticarrestdeficient-like1(yeast) | Nucleus |
| 65 | 213627_at | MAGED2 | melanomaantigenfamilyD,2 | PlasmaMembrane |
| 66 | 213256_at | MARCH3 | membrane-associatedringfinger(C3HC4)3 | Cytoplasm |
| 67 | 201555_at | MCM3 | minichromosomemaintenancecomplexcomponent3 | Nucleus |
| 68 | 214077_x_at | MEIS4 | Meishomeobox3pseudogene1 | unknown |
| 69 | 210605_s_at | MFGE8 | milkfatglobule-EGFfactor8protein | ExtracellularSpace |
| 70 | 212715_s_at | MICAL3(includesEG:57553) | microtubuleassociatedmonoxygenase,calponinandLIMdomaincontaining3 | Cytoplasm |
| 71 | 209708_at | MOXD1 | monooxygenase,DBH-like1 | Cytoplasm |
| 72 | 201761_at | MTHFD2 | methylenetetrahydrofolatedehydrogenase(NADP+dependent)2,methenyltetrahydrofolatecyclohydrolase | Cytoplasm |
| 73 | 212509_s_at | MXRA7 | matrix-remodellingassociated7 | unknown |
| 74 | 204798_at | MYB(includesEG:4602) | v-mybmyeloblastosisviraloncogenehomolog(avian) | Nucleus |
| 75 | 202431_s_at | MYC | v-mycmyelocytomatosisviraloncogenehomolog(avian) | Nucleus |
| 76 | 210395_x_at | MYL4 | myosin,lightchain4,alkali;atrial,embryonic | Cytoplasm |
| 77 | 202555_s_at | MYLK | myosinlightchainkinase | Cytoplasm |
| 78 | 201970_s_at | NASP | nuclearautoantigenicspermprotein(histone-binding) | Nucleus |
| 79 | 202607_at | NDST1 | N-deacetylase/N-sulfotransferase(heparanglucosaminyl)1 | Cytoplasm |
| 80 | 208714_at | NDUFV1 | NADHdehydrogenase(ubiquinone)flavoprotein1,51kDa | Cytoplasm |
| 81 | 202115_s_at | NOC2L | nucleolarcomplexassociated2homolog(S.cerevisiae) | Nucleus |
| 82 | 209120_at | NR2F2 | nuclearreceptorsubfamily2,groupF,member2 | Nucleus |
| 83 | 212691_at | NUP188 | nucleoporin188kDa | Nucleus |
| 84 | 210415_s_at | ODF2 | outerdensefiberofspermtails2 | Cytoplasm |
| 85 | 202733_at | P4HA2 | prolyl4-hydroxylase,alphapolypeptideII | Cytoplasm |
| 86 | 213675_at | PARVA | Parvin, alpha | Cytoplasm |
| 87 | 209493_at | PDZD2 | PDZdomaincontaining2 | PlasmaMembrane |
| 88 | 212094_at | PEG10 | paternallyexpressed10 | Nucleus |
| 89 | 201080_at | PIP4K2B | phosphatidylinositol-5-phosphate4-kinase,typeII,beta | Cytoplasm |
| 90 | 209581_at | PLA2G16 | phospholipaseA2,groupXVI | Nucleus |
| 91 | 204835_at | POLA1 | polymerase(DNAdirected),alpha1,catalyticsubunit | Nucleus |
| 92 | 209632_at | PPP2R3A | proteinphosphatase2(formerly2A),regulatorysubunitB'',alpha | Nucleus |
| 93 | 203529_at | PPP6C | proteinphosphatase6,catalyticsubunit | Nucleus |
| 94 | 207401_at | PROX1 | prosperohomeobox1 | Nucleus |
| 95 | 209162_s_at | PRPF4 | PRP4pre-mRNAprocessingfactor4homolog(yeast) | Nucleus |
| 96 | 209161_at | PRPF4 | PRP4pre-mRNAprocessingfactor4homolog(yeast) | Nucleus |
| 97 | 210832_x_at | PTGER3 | prostaglandinEreceptor3(subtypeEP3) | PlasmaMembrane |
| 98 | 208131_s_at | PTGIS | prostaglandinI2(prostacyclin)synthase | Cytoplasm |
| 99 | 204201_s_at | PTPN13 | proteintyrosinephosphatase,non-receptortype13(APO-1/CD95(Fas)-associatedphosphatase) | Cytoplasm |
| 100 | 203329_at | PTPRM | proteintyrosinephosphatase,receptortype,M | PlasmaMembrane |
| 101 | 200677_at | PTTG1IP | pituitarytumor-transforming1interactingprotein | Nucleus |
| 102 | 206499_s_at | RCC1(includesEG:1104) | regulatorofchromosomecondensation1 | Nucleus |
| 103 | 208492_at | RFXAP | regulatoryfactorX-associatedprotein | Nucleus |
| 104 | 200012_x_at | RPL21(includesEG:6144) | ribosomalproteinL21 | Cytoplasm |
| 105 | 200933_x_at | RPS4X | ribosomalproteinS4,X-linked | Cytoplasm |
| 106 | 201585_s_at | SFPQ | splicingfactorproline/glutamine-rich(polypyrimidinetractbindingproteinassociated) | Nucleus |
| 107 | 204688_at | SGCE | sarcoglycan,epsilon | PlasmaMembrane |
| 108 | 209402_s_at | SLC12A4 | solutecarrierfamily12(potassium/chloridetransporters),member4 | PlasmaMembrane |
| 109 | 204394_at | SLC43A1 | solutecarrierfamily43,member1 | PlasmaMembrane |
| 110 | 205398_s_at | SMAD3 | SMADfamilymember3 | Nucleus |
| 111 | 204240_s_at | SMC2 | structuralmaintenanceofchromosomes2 | Nucleus |
| 112 | 205443_at | SNAPC1 | smallnuclearRNAactivatingcomplex,polypeptide1,43kDa | Nucleus |
| 113 | 202200_s_at | SRPK1 | SFRSproteinkinase1 | Nucleus |
| 114 | 214882_s_at | SRFS2 | splicingfactor,arginine/serine-rich2 | Nucleus |
| 115 | 205016_at | TGFA | transforminggrowthfactor,alpha | ExtracellularSpace |
| 116 | 201042_at | TGM2 | transglutaminase2(Cpolypeptide,protein-glutamine-gamma-glutamyltransferase) | Cytoplasm |
| 117 | 203092_at | TIMM44 | translocaseofinnermitochondrialmembrane44homolog(yeast) | Cytoplasm |
| 118 | 203167_at | TIMP2 | TIMPmetallopeptidaseinhibitor2 | ExtracellularSpace |
| 119 | 210987_x_at | TPM1 | tropomyosin1(alpha) | Cytoplasm |
| 120 | 210986_s_at | TPM1 | tropomyosin1(alpha) | Cytoplasm |
| 121 | 203148_s_at | TRIM14 | tripartitemotif-containing14 | Cytoplasm |
| 122 | 203868_s_at | VCAM1 | vascularcelladhesionmolecule1 | PlasmaMembrane |
| 123 | 209822_s_at | VLDLR | verylowdensitylipoproteinreceptor | PlasmaMembrane |
| 124 | 203112_s_at | WHSC2 | Wolf-Hirschhornsyndromecandidate2 | Nucleus |
| 125 | 216022_at | Hs.669937 | Clone DKFZp564I153 |  |
