# Supplementary material for: Multi-Gene Expression Predictors of Single Drug Responses to Adjuvant Chemotherapy in Ovarian Carcinoma: Predicting Platinum Resistance
Source: PLoS One. 2012 Feb 10;7(2):e30550. doi: 10.1371/journal.pone.0030550 (PMC3277593; doi:10.1371/journal.pone.0030550)
Supplement: Table S2 — Performance of COXEN Single and Combination Predictors at the Youden cutoff. (DOC) [file pone.0030550.s005.doc]

**Supplementary Table S2. Performance of COXEN Single and Combination Predictors at the Youden cutoff.** The sensitivity, specificity, positive predictive value (PPV), and negative predictive value (NPV) were derived at the Youden cutoff values on an independent patient set of 119 cases.

| ***Compound*** | ***Data (Res, Nonres)*** | ***Sensitivity*** | ***Specificity*** | ***PPV*** | ***NPV*** | ***Youden*** |
| --- | --- | --- | --- | --- | --- | --- |
| **Carboplatin** | Dressman (85, 34) | 0.871(74/85)  (0.799-0.941) | 0.441 (15/34)  (0.274-0.608) | 0.796 (74/93)  (0.714-0.878) | 0.577 (15/26)  (0.387-0.767) | 0.3117 |
|  | UVA-55 (32, 23) | 0.875 (28/32)  (0.760-0.989) | 0.347 (8/23)  (0.153-0.542) | 0.651 (28/43)  (0.508-0.793) | 0.667 (18/28)  (0.400-0.933) | 0.223 |
| **Paclitaxel** | Dressman (85, 34) | 0.835 (71/85) (0.756-0.914) | 0.353 (12/26) (0.192-0.514) | 0.763 (71/93) (0.677-0.850) | 0.462 (12/26) (0.270-0.653) | 0.188 |
|  | UVA-55 (28, 23) | 0.821 (23/28)  (0.680-0.963) | 0.261 (6/23)  (0.081-0.440) | 0.575 (23/40)  (0.422-0.728) | 0.545 (6/11)  (0.251-0.839) | 0.082 |
| **Carbo/Tax** | Dressman (85, 34) | 0.835 (71/85) (0.756-0.914) | 0.412 (14/34) (0.246-0.577) | 0.780 (71/91) (0.695-0.865) | 0.5 (14/28) (0.315-0.685) | 0.247 |
|  | UVA-55 (28, 23) | 0.857 (24/28)  (0.727-0.897) | 0.348 (8/23)  (0.463-0.768) | 0.615 (24/39)  (0.462-0.768) | 0.667 (8/12)  (0.400-0.933) | 0.205 |
