# Supplementary material for: Multi-Gene Expression Predictors of Single Drug Responses to Adjuvant Chemotherapy in Ovarian Carcinoma: Predicting Platinum Resistance
Source: PLoS One. 2012 Feb 10;7(2):e30550. doi: 10.1371/journal.pone.0030550 (PMC3277593; doi:10.1371/journal.pone.0030550)
Supplement: Table S3 — A multivariate logistic regression analysis both with COXEN and other clinical variables on the UVA-55 cohort. (DOC) [file pone.0030550.s006.doc]

**Supplementary Table S3. A multivariate logistic regression analysis both with COXEN and other clinical variables on the UVA-55 cohort**

| ***Name*** | ***Estimate*** | ***Std. Error*** | ***z value*** | ***Pr(>|z|)*** |
| --- | --- | --- | --- | --- |
| (Intercept) | 16.579 | 2399.546 | 0.007 | 0.994 |
| COXEN | 3.038 | 1.530 | 1.985 | **0.047** |
| STAGE | -16.093 | 2399.545 | -0.007 | 0.995 |
| AGE | -0.013 | 0.045 | -0.279 | 0.780 |
| DEBULKING | -0.135 | 0.887 | -0.153 | 0.879 |
| RACE | -1.133 | 1.520 | -0.746 | 0.456 |
