# Supplementary material for: Multi-Gene Expression Predictors of Single Drug Responses to Adjuvant Chemotherapy in Ovarian Carcinoma: Predicting Platinum Resistance
Source: PLoS One. 2012 Feb 10;7(2):e30550. doi: 10.1371/journal.pone.0030550 (PMC3277593; doi:10.1371/journal.pone.0030550)
Supplement: Text S1 — (DOC) [file pone.0030550.s007.doc]

**Supplementary Methods**

**The “Co-eXpression ExtrapolatioN” (COXEN) algorithm**

To calculate the *in vitro*-COXEN prediction scores, the gene expression data were normalized using RMAExpress 1.0 software and standardized by subtracting the mean and dividing the result by the standard deviation for each probe level measurement. The COXEN algorithm is composed of six distinct steps. The end result is what we term the “COXEN Score,” which reflects the predicted sensitivity of a particular cell line or human tumor to the specific drug being evaluated by the algorithm. Generically, the steps for prediction of a drug’s activity in cells belonging to some Set 2 on the basis of its activity pattern in different cells of some Set 1 are as follows:

*Step 1.* Experimentally determine the drug’s pattern of activity in cells of Set 1.

*Step 2.* Experimentally measure molecular characteristics of the cells in Set 1.

*Step 3.* Select a subset of those molecular characteristics that most accurately predicts the drug’s activity in cell Set 1 (“chemosensitivity signature” selection).

*Step 4.* Experimentally measure the same molecular characteristics of the cells in Set 2.

*Step 5.* Among the molecular characteristics selected in step 3, identify a subset that shows a strong pattern of “co-expression extrapolation” between cell Sets 1 and 2.

*Step 6.* Use a multivariate algorithm to predict the drug’s activity in Set 2 cells on the basis of the drug’s activity pattern in Set 1 and the molecular characteristics of Set 2 selected in step 5. The output of the multivariate analysis is a COXEN Score.

In our first application of the COXEN algorithm, for example, Cell Sets 1 and 2 were the NCI-60 and one of the training set of human breast cancer patients (n=251; Miller et al. An expression signature for p53 status in human breast cancer predicts mutation status, transcriptional effects, and patient survival. Proc Natl Acad Sci USA, 2005; BR-251); the Step 1 drug activities were those assessed by the DTP in the NCI-60; the “molecular characteristics” in Steps 2 and 4 were transcript expression levels, as assessed using Affymetrix HG-U133A microarrays; the algorithm in Step 3 was “Significance Analysis of Microarrays (SAM)” or similar statistical testing for differential expression; Step 5 is a novel “co-expression extrapolation” algorithm we developed. Step 6 was a refined classification algorithm, “Misclassification-Penalized Posterior” (MiPP) which we recently introduced for selection of the best mathematical “models” for such predictions (Soukup et al, Robust classification modeling on microarray data using misclassification penalized posterior. Bioinformatics, 2005). MiPP generates the final COXEN Score. As will be discussed below, COXEN predictions for both cell line and clinical trial drug responses were prospectively and independently validated.

## Identification of candidate “chemosensitivity biomarkers” in the NCI-60 panel (Step 3)

For each compound in the public NCI-60 drug database, we identified each top 20~35% most sensitive and most resistant NCI-60 cell lines. Using slightly different percent cutoffs did not change the ultimate results appreciably (data not shown). After selection of NCI-60 cells sensitive and resistant to each of the two compounds, we used the “Significance Analysis of Microarrays” (SAM) test with false discovery rate (FDR) 0.1 to identify microarray probe sets expressed differentially between the two cell subsets. Those probe sets can be thought of as candidate “chemosensitivity biomarkers” based on the NCI-60 data.

**Identification of co-expression extrapolation signatures (Step 5)**

To parameterize each probe’s co-expression relationships between two studies mathematically, we calculated a “co-expression extrapolation coefficient (CEEC),” rc(j), for each probe j as follows: Using the probe expression data, we constructed two correlation matrices (of dimension n x n) for the set of n candidate chemosensitivity probes. The two correlation matrices, one for the NCI-60, the other for the training patient set BR-251, were evaluated as U = [Uij]_nxn_ and V = [Vij]_nxn_,, where Uij and Vij are the correlation coefficients between probes i and j in the NCI-60 and BR-251, respectively. Then, *rc(j)* is defined as

$$rc(j)=\frac{\sum_{k=1}^{n} (U_{kj}-\mathop{U_{j}}\limits^{-} \mathop{)(V_{kj}-\mathop{V_{j})}\limits^{-} }\limits}{\sqrt{\sum_{k=1}^{n} (U_{kj}-\mathop{U}\limits^{-} _{j}\mathop{)^{2}}\limits}\sqrt{\sum_{k=1}^{n} (V_{kj}-\mathop{V_{j}}\limits^{-} \mathop{)^{2}}\limits}}$$

where $\mathop{U_{j}}\limits^{-}$and $\mathop{V_{j}}\limits^{-}$ are the mean correlation coefficients of the column-j correlation coefficient vectors for the NCI-60 and BR-251. rc reflects the degree of co-expression extrapolation of probe j with the set of n probes between the NCI-60 and BR-251 panels. If rc(j) exceeded a cut-off criterion (e.g., 98th percentile of the corresponding random distribution generated by randomly shuffling the probe identities between the two sets), probe j was selected as a probe for co-expression extrapolation between the two panels. Since probe j was selected from the set of n candidate chemosensitivity predictors, it had that pharmacological characteristic as well. Note that CEEC will be high if a probe’s co-expression network relationships with the other probes on the first set (i.e. NCI-60) are concordant with those of the second set (i.e. BR-251). The co-expression extrapolation coefficient (CEEC), rc(j), is thus simply the correlation coefficient between column vectors $\mathop{U_{j}}\limits$and $\mathop{V_{j}}\limits$.

**Development of chemosensitivity prediction models for the *in vitro* COXEN GEM (Step 6)**

We searched among those candidate biomarkers obtained from steps 1-5 for ones that would form optimal parsimonious models for prediction of the compound’s activity. For that purpose, we used the “Misclassification-Penalized Posterior” (MiPP) algorithm. In brief, MiPP is based on stepwise incremental classification modeling for discovery of the most parsimonious prediction models. It includes double cross-validated evaluation for each trained prediction model. Model training can be performed using any of several different classification algorithms such as linear discriminant analysis (LDA), quadratic discriminant analysis (QDA), support vector machine (SVMs) learning, or logistic regression. In the current study, we used LDA for most of the applications. In double cross-validation, the first cross-validation is based on random splitting of the whole data set into a training set and an independent test set for external model validation; the second is an n-fold cross-validation on the training set to avoid the pitfalls of a large-screening search and to obtain the most parsimonious optimal prediction models. Independent splits of the data result in multiple prediction models. The multiple models are then re-evaluated using a large number (e.g., 1000) of random splits of test and training sets to obtain confidence bounds on the accuracy of prediction. On the basis of those confidence bounds, the prediction performance and mean misclassification error rates (ER) are obtained for each of the candidate prediction models. The final prediction of a cell line as “sensitive” or “resistant” is determined by the cell’s (posterior) classification probability (CP) from each LDA prediction model. If CP > 0.5 is based on the top 3-5 prediction models, the cell line is considered sensitive; if not, it is considered resistant. We found that optimal MiPP prediction models were often obtained with only a small number of probes, e.g. three or four probes, among the ones identified in Step 5.

**Combination-drug GEM**

Individual compound GEMs are combined to generate the prediction model for each combination chemotherapy under the assumption that the individual compounds in the combination act independently. That is, prediction of combination drug efficacy was obtained based on the final single-drug prediction models, directly utilizing each cell line’s classification probabilities from these models. That is, assuming two different drug compounds acted independently, the combination chemosensitivity probability **P_AB_** of their combination treatment was derived as:

*1 –* *P_A_[resistant for drug A] x P_B_[resistant for drug B].*

Here **P_A_** and **P_B_** are the chemosensitivity response probabilities based on the prediction models for compound A and B, respectively. Importantly, this model development and training *did not* use any clinical information or microarray data from the test sets used for GEM evaluation, thus maintaining strict independence between training and test data sets.
